# SK2 channels in cerebellar Purkinje cells contribute to excitability modulation in motor-learning–specific memory traces

**DOI:** 10.1371/journal.pbio.3000596

**Published:** 2020-01-06

**Authors:** Giorgio Grasselli, Henk-Jan Boele, Heather K. Titley, Nora Bradford, Lisa van Beers, Lindsey Jay, Gerco C. Beekhof, Silas E. Busch, Chris I. De Zeeuw, Martijn Schonewille, Christian Hansel

**Affiliations:** 1 Department of Neurobiology, University of Chicago, Chicago, Illinois, United States of America; 2 Department of Neuroscience, Erasmus MC, Rotterdam, The Netherlands; 3 Netherlands Institute for Neuroscience, Royal Academy of Sciences, Amsterdam, The Netherlands; ICM - Institut du Cerveau et de la Moelle épinière Hôpital Pitié-Salpêtrière 47, bd de l’Hôpital, FRANCE

## Abstract

Neurons store information by changing synaptic input weights. In addition, they can adjust their membrane excitability to alter spike output. Here, we demonstrate a role of such “intrinsic plasticity” in behavioral learning in a mouse model that allows us to detect specific consequences of absent excitability modulation. Mice with a Purkinje-cell–specific knockout (KO) of the calcium-activated K^+^ channel SK2 (L7-SK2) show intact vestibulo-ocular reflex (VOR) gain adaptation but impaired eyeblink conditioning (EBC), which relies on the ability to establish associations between stimuli, with the eyelid closure itself depending on a transient suppression of spike firing. In these mice, the intrinsic plasticity of Purkinje cells is prevented without affecting long-term depression or potentiation at their parallel fiber (PF) input. In contrast to the typical spike pattern of EBC-supporting zebrin-negative Purkinje cells, L7-SK2 neurons show reduced background spiking but enhanced excitability. Thus, SK2 plasticity and excitability modulation are essential for specific forms of motor learning.

## Abstract 2

This study shows that cerebellum-dependent motor learning involves an adjustment of membrane excitability in Purkinje cells via modulation of SK2-type K+ channels; this "intrinsic plasticity" requirement can be separated from changes in synaptic strength.

## Introduction

The association of learning with changes in the membrane excitability of neurons was first described in invertebrate mollusks such as *Hermissenda* and *Aplysia* [1–5] but is similarly found in the vertebrate hippocampus [6–8] and in the cerebellar cortex and nuclei [9–12]. Is there a “memory from the dynamics of intrinsic membrane currents,” as previously suggested by Eve Marder and colleagues [13]? Despite significant progress in the field, it has been difficult to comprehensively describe the cellular mechanisms underlying vertebrate behavioral learning. This also holds for relatively simple forms of cerebellum-dependent motor learning, such as delay eyeblink conditioning (EBC) [14, 15] and adaptation of the vestibulo-ocular reflex (VOR) [16–18]. An important step forward has been the realization that we need to abandon attempts to link even simple behaviors to one specific type of cellular plasticity and instead appreciate learning as a result of multiple distributed, yet synergistic, plasticity events [19–22]. The question that we want to address here is whether cell-autonomous changes in membrane excitability are indeed a component of such plasticity networks and whether this intrinsic component is essential for the proper execution of a behavioral memory task. We chose cerebellum-dependent forms of motor learning, VOR gain adaptation and delay EBC, as examples of behavioral learning to study because both are associated with changes in simple spike firing, indicating that excitability adjustment is part of their respective memory engrams, or “mnemic traces” [23].

VOR adaptation is the adjustment of an eye movement reflex in response to head rotation, aimed at optimizing vision and driven by retinal slip. VOR gain increase, the adaptive amplification of the reflex, has been linked to an increase in simple spike firing rates of Purkinje cells [18]. In mice with a Purkinje-cell–specific knockout (KO) of protein phosphatase 2B (L7-PP2B mice) or GluA3 (L7-GluA3 mice), both synaptic and intrinsic potentiation are prevented, indeed resulting in a decrease of VOR gain upon gain increase training [24, 25].

EBC is a form of associative learning in which animals develop a protective eyelid closure response to a neutral conditioned stimulus (CS), such as a brief light flash or a tone, when the CS is repeatedly paired with an unconditioned stimulus (US), typically a periorbital air puff. In the “delay” version of the task, the onset of the CS precedes that of the US with a precisely timed interval, and either both stimuli coterminate or the CS ends after the US, thus avoiding a gap between the two stimuli (for review, see [26]). Bernard Schreurs and colleagues reported that EBC learning in rabbits is associated with an increase in the excitability of Purkinje cell dendrites that was observed in slices prepared from cerebellar lobule HVI 24 hours or a month after paired, but not with pseudoconditioning [9, 10]. Among other measures, the authors reported a reduction in the amplitude of the afterhyperpolarization (AHP) following depolarizing current steps [10]. We have been able to generally confirm this observation in mice: 48 hours following EBC conditioning, we observed a reduction in the amplitude of the AHP following a parallel fiber (PF)-excitatory postsynaptic potential (EPSP) train in Purkinje cells at the base of the primary fissure near lobules V/HVI [11], which has now been established as one of the cerebellar areas that drives EBC learning in mice [27, 28].

An observation that seems to be at odds with the findings from these ex vivo studies is that the eyeblink conditioned response (CR) is well-predicted by a suppression in Purkinje cell simple spike firing that may last for several hundreds of milliseconds [28–31]. This spike suppression matches predictions from the Albus–Ito theory that inhibition of Purkinje cell activity leads to a disinhibition of target neurons in the cerebellar nuclei [27, 32, 33]. Indeed, as originally envisioned, long-term synaptic depression (LTD) at PF synapses onto Purkinje cells [34] has the potential to contribute to this spike suppression in EBC because a reduction of excitatory input will minimize an increase in simple spike activity when these PFs are activated [16, 35, 36]. Because individual Purkinje cells can undergo PF synaptic plasticity timed to a wide range of PF-climbing fiber (CF) intervals, such plasticity may contribute to the firing rate reduction over an extended time period [37]. A more immediate effector of simple spike suppression is active inhibition evoked by molecular layer interneurons (MLIs) that receive enhanced excitatory drive from PFs [20, 28, 36]. LTD and enhanced MLI inhibition probably act synergistically to optimize simple spike suppression. In isolation, LTD is not essential for EBC [38, 39], but a contribution of LTD to proper EBC motor learning is revealed in vivo when compensation by MLIs is absent [22].

The observation of enhanced excitability is not easily explained in light of the seemingly contradictory need for simple spike suppression. However, the available data make a strong case that excitability regulation in general plays a central role in EBC. To investigate such a role of SK2-dependent excitability control in cerebellar motor learning, we generated and tested mice with a Purkinje-cell–specific KO of SK2 channels (L7-SK2). This type of calcium-dependent K^+^ channel, which is the only SK isoform expressed in Purkinje cells [40], has been shown to contribute to the AHP following spike bursts in Purkinje cells [41, 42]. Activity-dependent intrinsic plasticity (increase in excitability) in Purkinje cells in vitro is accompanied by a reduction in the AHP amplitude, is mimicked and occluded by the SK channel blocker apamin, and is absent in SK2 channel KO mice [43, 44]). Similarly, in our recent EBC experiments, we found occlusion of intrinsic plasticity in Purkinje cells from paired-conditioned mice but fully expressed intrinsic plasticity in Purkinje cells from pseudoconditioned mice [11]. In the present study, mice were investigated not only at the cell physiological and histological level but also at the systems behavioral level, employing a large variety of learning tasks that are known to depend on specific modules of the olivocerebellar system [45]. We found that in L7-SK2 mice, EBC conditioning is selectively impaired, whereas other forms of motor learning that do not mainly rest on simple spike suppression, such as VOR gain adaptation, are unaffected or even improved. These observations suggest a critical contribution of SK2-mediated excitability modulation and plasticity to the formation of specific cerebellar motor engrams.

## Results

### L7-SK2 mice lack SK2 selectively in Purkinje cells but show normal histology

**Fig 1:**
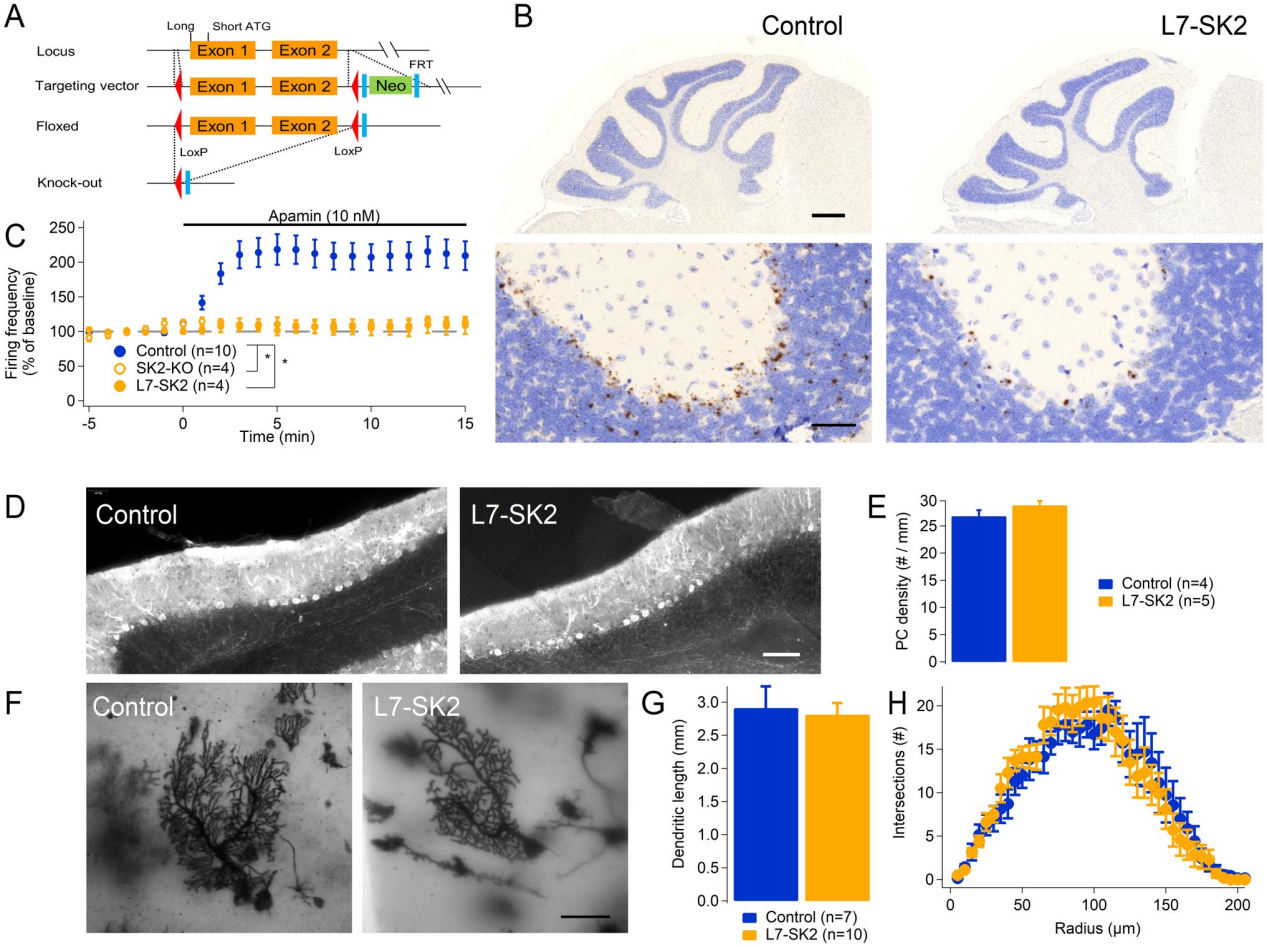
The L7-SK2 mutant: Generation and morphology. (A) A conditional mutation was generated with the CRE-Lox system introducing LoxP sites flanking the first two coding exons of the *Kcnn2* locus, coding for SK2 (both long and short isoforms). A downstream Neo cassette—flanked by FRT sequences—was used in the targeting vector as a reporter during the selection of recombinant embryonic stem cell clones and removed by crossing mutant mice with a mouse strain with constitutive expression of the FLP recombinase. The resulting “floxed” mouse strain was crossed with L7-CRE mice, expressing the CRE recombinase in a PC-specific way to obtain PC-specific KO (L7-SK2). (B) In situ hybridization performed by RNAscope revealed a virtually complete absence of the *Kcnn2* mRNA in the PC layer (right panels) compared to floxed control littermates. The gross morphology and foliation did not show evident alterations (scale bar: 500 μm and 50 μm in the upper and lower panels, respectively). (C) The absence of functional SK channels was tested for sensitivity to the selective SK channel agonist apamin (10 nM). The graph shows the firing frequency of PCs evoked by a somatic depolarizing current injection of fixed amplitude before and after the administration of the drug in the bath (a negative bias current was injected to prevent spontaneous firing). Only control cells increased their firing frequency in response to the drug, while both SK2-KO and L7-SK2 did not show any significant change in firing frequency, confirming the absence of SK2 channels and of other apamin-sensitive channels. (D) PC density assessed on sections stained with anti-calbindin antibody (scale bar: 100 μm) was comparable in L7-SK2 mice and control littermates. (E) Bar graph showing the PC density in control and L7-SK2 mice. (F) Golgi-stained PCs in control (left) and L7-SK2 mice (right; scale bar: 50 μm). (G) Bar graph showing the total length of the PC dendritic tree in control and L7-SK2 mice. (H) Sholl analysis showed no significant difference in the morphology of PCs from L7-SK2 and control mice. *p < 0.05. See also S1 Fig. CRE, causes recombination; FLP, flippase; FRT, FLP recombinase target; *Kcnn2*, potassium calcium-activated channel subfamily N member 2; KO, knockout; LoxP, locus of X-over of P1; PC, Purkinje cell.

We generated a Purkinje-cell–specific SK2 KO mouse (“L7-SK2” mouse Fig 1A) with locus of X-over of P1 (LoxP) sites flanking the first two coding exons of the potassium calcium-activated channel subfamily N member 2 (*Kcnn2*) gene and crossed this line with a second line expressing the LoxP-recognizing recombinase CRE under the Purkinje-cell–specific promoter Purkinje cell protein 2 (Pcp2)/L7 [46, 47]. In situ hybridization shows that *Kcnn2* mRNA is expressed in the Purkinje cell layer of control littermates but is virtually absent in L7-SK2 mice (Fig 1B). These sections also show that there are no major differences in foliation of the cerebellar cortex between control and L7-SK2 mice (Fig 1B). Using the previously reported constitutive SK2-KO as a null reference [48], a pharmacological analysis of the sensitivity to the SK channel blocker apamin demonstrated that, while this toxin causes a dramatic increase of evoked spike frequency in control Purkinje cells, it does not affect evoked Purkinje cell firing in L7-SK2 or in SK2-KO mice, confirming the absence of functional SK channels in L7-SK2 Purkinje cells (Fig 1C; relative increase in spike frequency at 10–15 min: control, 211.0 ± 20.2%, *n* = 10; L7-SK2: 106.0 ± 11.4%, *n* = 4; SK2-KO: 108.0 ± 5.7%, *n* = 4; Kruskal–Wallis: *p* = 0.0018; post hoc comparison: *p* = 0.013 for control versus L7-SK2, *p* = 0.013 for control versus SK2-KO, *p* = 1.00 for L7-SK2 versus SK2-KO). Immunostaining for the Purkinje-cell–specific marker calbindin showed that the density of Purkinje cells is not different between control (26.8 ± 11 cells/mm, *n* = 4) and L7-SK2 mice (28.8 ± 0.8 cells/mm, *n* = 5; *p* = 0.118; Fig 1D and 1E). This was also observed in general SK2-KO mice (S1 Fig), confirming that SK2 does not play a major role in Purkinje cell generation or degeneration [44]. Measuring the total length and complexity of the dendritic arbor by Golgi staining and subsequent Sholl analysis in the new L7-SK2 strain (Fig 1F–1H), we did not observe any difference in dendritic length between control (2.9 ± 0.3 mm, *n* = 7) and L7-SK2 mice (2.8 ± 0.2 mm, *n* = 10; *p* = 0.97; Fig 1G), nor any difference in dendritic branching complexity as tested by Sholl analysis (genotype effect: F[1, 15] = 0.032, *p* = 0.86; genotype × radius interaction: F[40, 600] = 0.60, *p* = 0.98; Fig 1H), again confirming that SK2 channels do not play a major role in Purkinje cell morphogenesis.

### Membrane excitability of L7-SK2 Purkinje cells is enhanced without affecting basic synaptic transmission

**Fig 2:**
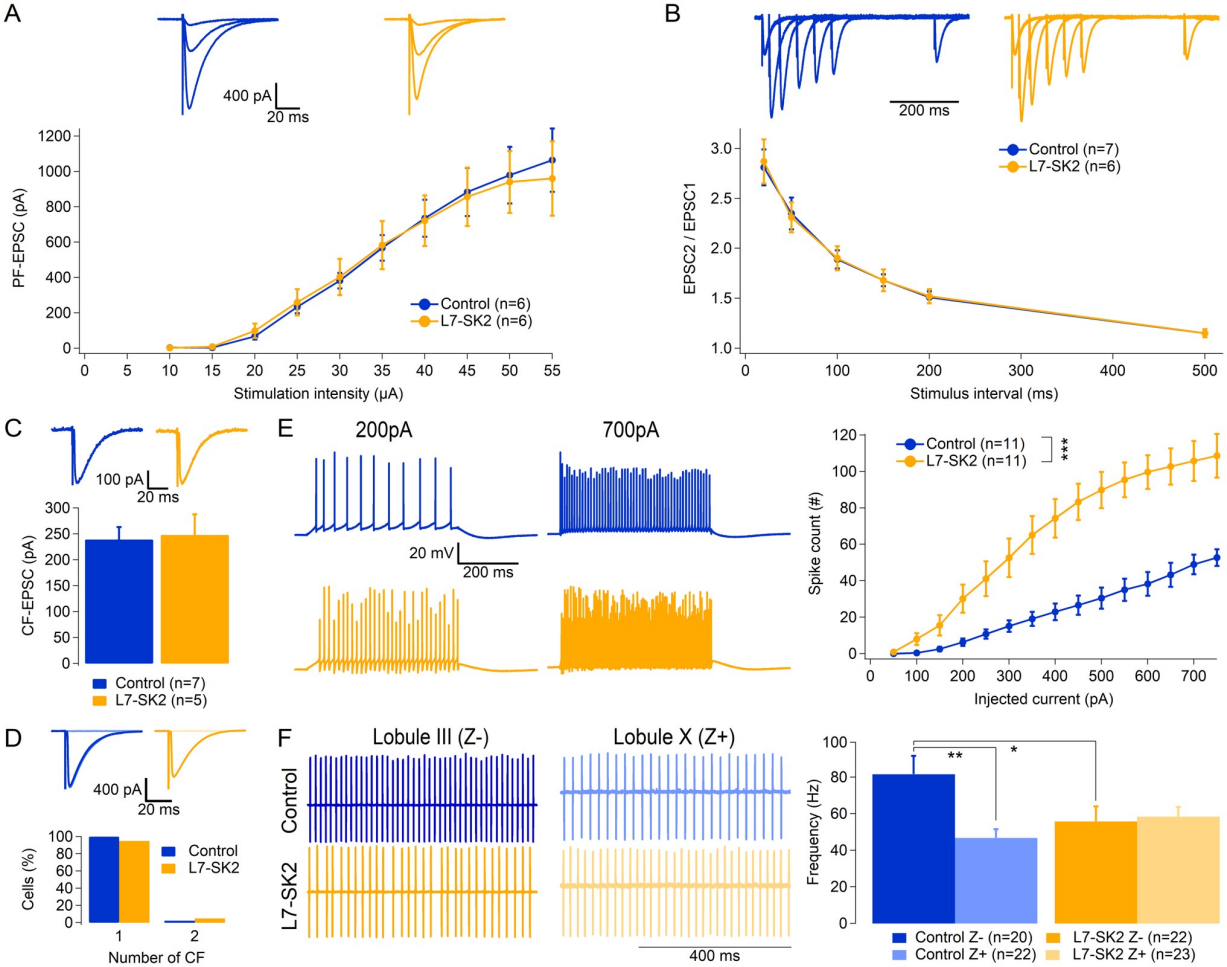
Intrinsic excitability is increased in L7-SK2 mice. (A) PF strength was comparable in L7-SK2 and control mice, as assessed recording PF-EPSCs with increasing stimulation intensities, showing no significant differences (upper panel: representative traces, lower panel: PF-EPSC averaged per group at different stimulation intensities). (B) PF-EPSC PPR was comparable in L7-SK2 and control mice. It was calculated at increasing interpulse intervals as the ratio of the second EPSC over the first one, as a proxy of synaptic release probability (upper panel: representative traces, rescaled for each individual EPSC1; lower panel: PF-EPSCs averaged per group at different stimulation intensities). (C) CF-EPSCs were comparable in L7-SK2 and control mice (upper panel: representative traces, Vm = 0 mV; lower panel: CF-EPSCs averaged per group). (D) The number of CF inputs was comparable in both groups with a virtual absence of multi-innervation (upper panel: representative traces, Vm = −20 mV; lower panel: distribution of the number of CF inputs). (E) Purkinje cell excitability was increased in L7-SK2 mice, as assessed by the number of spikes evoked by a somatic depolarizing current injection of increasing amplitude (left panel: representative traces at 200 and 700 pA, right panel: spike count averaged per group per current amplitude). (F) Spontaneous simple spike firing rates in zebrin-negative (Z−; lobule III) and zebrin-positive (Z+; lobule X) Purkinje cells. *p < 0.05; **p < 0.01; ***p < 0.001. CF, climbing fiber; EPSC, excitatory postsynaptic current; PF, parallel fiber; PPR, pair-pulse ratio.

In order to test the cellular consequences of the SK2 knockout, we first examined the basic physiological properties of Purkinje cells. The excitatory postsynaptic current (EPSC) at PF to Purkinje cell synapses measured at increasing stimulus intensity was comparable between L7-SK2 mice and their control littermates (Fig 2A; genotype effect: F[1, 9] = 0.11, *p* = 0.75; stimulation intensity × genotype interaction: F[9, 81] = 0.26, *p* = 0.98, *n* = 6 cells per genotype), indicating similarly functional connectivity. The paired-pulse ratio (EPSC2/EPSC1) measured at interstimulus intervals ranging from 20–500 ms was also comparable between the two groups, suggesting that the Purkinje-cell–specific mutation does not affect presynaptic release properties at PF to Purkinje cell synapses (Fig 2B; genotype effect: F[1, 10] = 0.077, *p* = 0.79; interval × genotype interaction: F[5, 50] = 0.21, *p* = 0.96; *n* = 7 and 6, respectively, for control and L7-SK2). We then tested synaptic transmission at CF to Purkinje cell synapses, which convey error signals and are crucial for PF-LTD induction. CF-EPSC amplitudes were comparable between control (239.2 ± 24.0 pA; *n* = 7) and L7-SK2 Purkinje cells (248.2 ± 39.6 pA; *n* = 5; *p* = 0.81; Fig 2C). There was also no difference in the developmental elimination of CFs between Purkinje cells from ≥P21 L7-SK2 mice and wild-type (WT) controls (P21–136; WT: 1 CF input in 13 out of 13 Purkinje cells from 3 mice; L7-SK2: 1 CF input in 18 out of 19 Purkinje cells from 3 mice, one Purkinje cell was innervated by two CF inputs; Fisher exact test: *p* = 1.00; Fig 2D). Together, these data show that the Purkinje-cell–specific deletion of SK2 channels does not affect the development of synaptic connectivity in Purkinje cells. Membrane excitability was tested at near-physiological temperature (32 °C–34 °C) by injection of depolarizing current pulses. SK2 deletion resulted in a significant increase in excitability (Fig 2E; genotype effect F[1, 20] = 27.85, *p* < 0.001; injected current × genotype interaction: F[14, 280] = 9.72, *p* < 0.001, *n* = 11 cells per group), compatible with the hyperpolarizing function played by these K^+^ channels [49].

Finally, we characterized spontaneous spike firing rates of Purkinje cells in control and L7-SK2 mice. In WT controls, the spontaneous simple spike firing activity differs by association with zebrin-positive or zebrin-negative zones. Purkinje cells in zebrin-negative zones have higher spike firing rates than those in zebrin-positive zones [35]. Purkinje cells in the eyeblink zone near lobules V/HVI are largely zebrin-negative, but the separation is not as complete as in more anterior (e.g., lobules I–IV; zebrin-negative) or more posterior lobules (lobules IX and X; zebrin-positive) [35]. For this reason, we compared spike firing rates of Purkinje cells in control and L7-SK2 mice that are located in lobules III and X so as to reach spike firing rate homogeneity within these groups. We observed that the genotype alone had no significant effect on the simple spike frequency (two-way ANOVA genotype effect: *p* = 3.29, F[1, 83] = 0.964). However, there was a lobule effect (*p* = 0.028, F[1, 83] = 5.01): the spike frequency was higher in lobule III than in lobule X Purkinje cells in control mice (lobule III: 81.6 ± 10.0 Hz, *n* = 20; lobule X: 46.7 ± 4.7 Hz, *n* = 22; Least Significant Difference (LSD) post hoc test, *p* = 0.0012; Fig 2F). There was also a significant effect of the interaction between genotype and lobule (*p* = 0.011, F[1, 83] = 6.79): in L7-SK2 mice, lobule III Purkinje cells showed a significant reduction in firing frequency as compared to control mice (55.8 ± 8.2 Hz, *n* = 22; LSD post hoc test, *p* = 0.015), while in lobule X, they had comparable frequency levels (58.4 ± 5.3 Hz; *n* = 23) to lobule X Purkinje cells in control mice (LSD post hoc test, *p* = 0.246), as well as to lobule III Purkinje cells in L7-SK2 mice (*p* = 0.792). As a net effect, the original difference in spike firing rates between these zebrin-negative and zebrin-positive lobules in controls disappeared in L7-SK2 mice. These results suggest that the absence of SK channels in Purkinje cells does not cause a general increase in the spontaneous discharge rate, as might have been expected, but instead leads, in zebrin-negative Purkinje cells, to a significant reduction.

### Intrinsic plasticity, but not PF synaptic plasticity, is impaired in Purkinje cells of L7-SK2 mice

**Fig 3:**
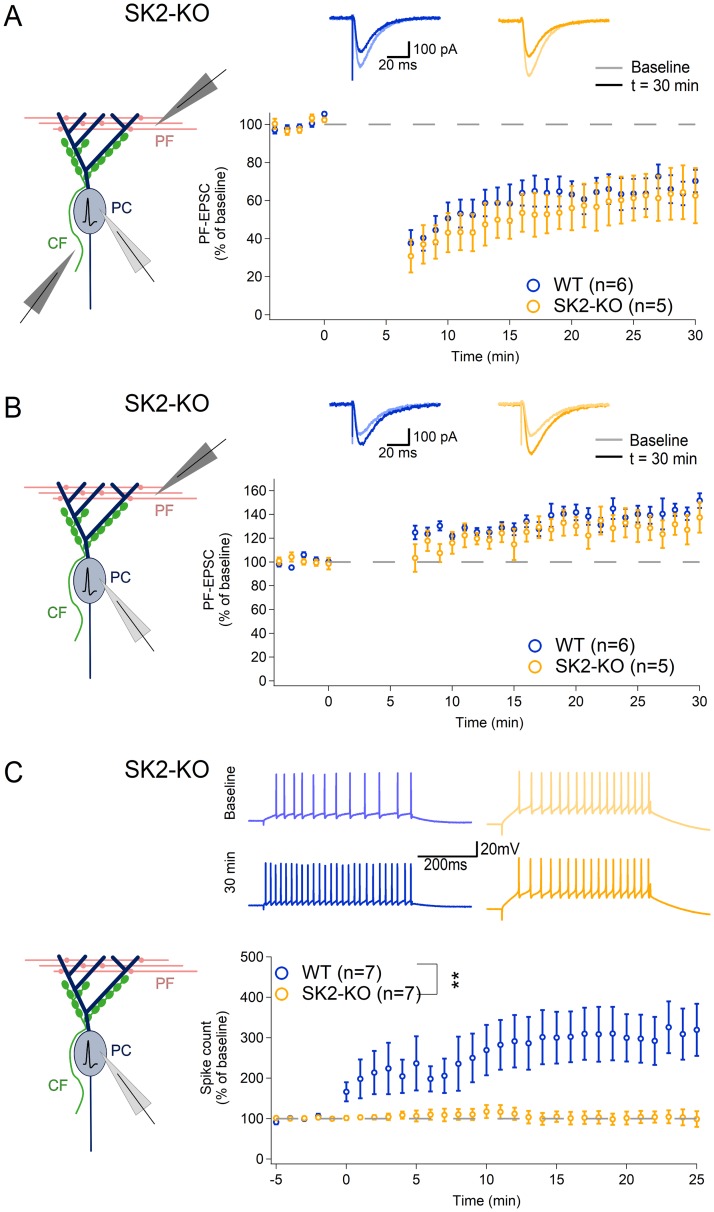
Intrinsic plasticity is selectively impaired in SK2-KO PCs. (A) LTD is induced at comparable levels at PF synapses in SK2-KO PCs and WT littermates (induction protocol: 1-Hz paired PF + CF single pulse, 5 min). Upper panel: representative traces of PF-EPSCs. Lower panel: time graph of PF-EPSCs (averaged per minute) before and after the induction (at 0 min). (B) LTP is induced at comparable levels at PF synapses in SK2-KO PCs and WT littermates (induction protocol: 1-Hz PF single pulse, 5 min). Upper panel: representative traces of PF-EPSCs. Lower panel: time graph of PF-EPSCs as in A. (C) Intrinsic plasticity cannot be induced in SK2-KO PCs by a somatic depolarization protocol (this panel is reproduced in a modified form from [44]). Upper panel: representative traces of spikes evoked by a somatic depolarizing current injection of fixed amplitude, before (upper) and after (lower) the induction protocol. Lower panel: time graph of spike count (averaged per minute) before and after the induction (at 0 min). **p < 0.01. CF, climbing fiber; EPSC, excitatory postsynaptic current; KO, knockout; LTD, long-term synaptic depression; PC, Purkinje cell; PF, parallel fiber; WT, wild type.

As previously demonstrated, intrinsic plasticity is absent from SK2-KO mice (Fig 3) [44]. At hippocampal Schaffer collateral–CA1 synapses, SK2 channel regulation has an impact on synaptic transmission and plasticity [50]. To evaluate whether similar effects exist in the cerebellum, we tested PF-LTD and long-term potentiation (LTP) first in the constitutive SK2-KO mice, then in L7-SK2 mice. In the general KOs, both LTD (SK2-KO: 62.1 ± 14.2%, *n* = 5; WT: 67.9 ± 6.7%, *n* = 6; EPSC amplitude measured at *t* = 26–30 min after tetanization; difference between groups: *p* = 1.00) and LTP (SK2-KO: 125.0 ± 11.2%, *n* = 5; WT: 143.6 ± 7.2%, *n* = 6; difference between groups: *p* = 0.36) were unaltered (Fig 3), showing that knocking out SK2 channels causes a selective impairment of intrinsic plasticity, without affecting synaptic plasticity.

**Fig 4:**
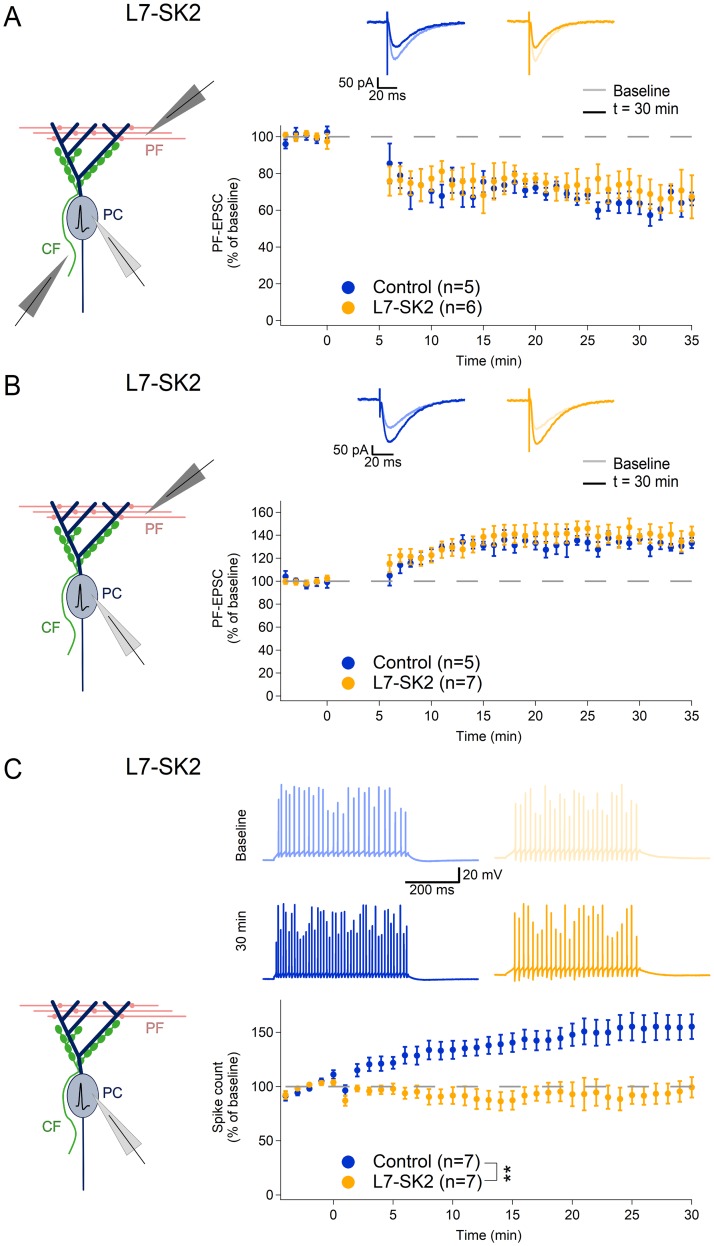
Intrinsic plasticity is selectively impaired in L7-SK2 PCs. (A) Similar to SK2-KO cells (Fig 3), L7-SK2 PCs express LTD at PF synapses at comparable levels to control littermates (induction protocol: 1-Hz paired PF + CF single pulse, 5 min). Upper panel: representative traces of PF-EPSCs. Lower panel: time graph of PF-EPSCs (averaged per minute) before and after the induction (at 0 min). (B) LTP is induced at comparable levels at PF synapses in L7-SK2 PCs and control littermates (induction protocol: 1-Hz PF single pulse, 5 min). Upper panel: representative traces of PF-EPSCs. Lower panel: time graph of PF-EPSCs as in A. (C) Intrinsic plasticity cannot be induced in L7-SK2 PCs by a somatic depolarization protocol. Upper panel: representative traces of spikes evoked by a somatic depolarizing current injection of fixed amplitude, before (upper) and after (lower) the induction protocol. Lower panel: time graph of spike count (averaged per minute) before and after the induction (at 0 min). **p < 0.01. CF, climbing fiber; EPSC, excitatory postsynaptic current; KO, knockout; LTD, long-term synaptic depression; LTP, long-term potentiation; PC, Purkinje cell; PF, parallel fiber.

On the basis of our observations in SK2-KO mice (Fig 3), we hypothesized that L7-SK2 mice would show a selective impairment of Purkinje cell intrinsic plasticity but unaffected PF synaptic plasticity. We indeed found no differences in LTP and LTD between L7-SK2 mice and their WT littermate controls (LTD—WT: 63.7 ± 4.3%, *n* = 5; L7-SK2: 72.8 ± 6.5%, *n* = 6; *p* = 0.27; Fig 4A. LTP—WT: 131.0 ± 4.9%, *n* = 5; L7-SK2: 143.2 ± 7.4%, *n* = 7; *p* = 0.29; Fig 4B). In contrast, intrinsic plasticity was absent from L7-SK2 mice (94.2 ± 9.5%, *n* = 7; WT: 155.0 ± 11.6%, *n* = 7; *p* = 0.010; Fig 4C), showing that the Purkinje-cell–specific SK2 mutation—just like the constitutive SK2 knockout—caused an impairment of intrinsic plasticity without altering synaptic plasticity. This selective impairment of intrinsic plasticity therefore makes L7-SK2 mice an excellent model to study the role of SK2-dependent control of Purkinje cell excitability in cerebellar learning independent from synaptic plasticity.

### Locomotion and motor performance in L7-SK2 mice

**Fig 5:**
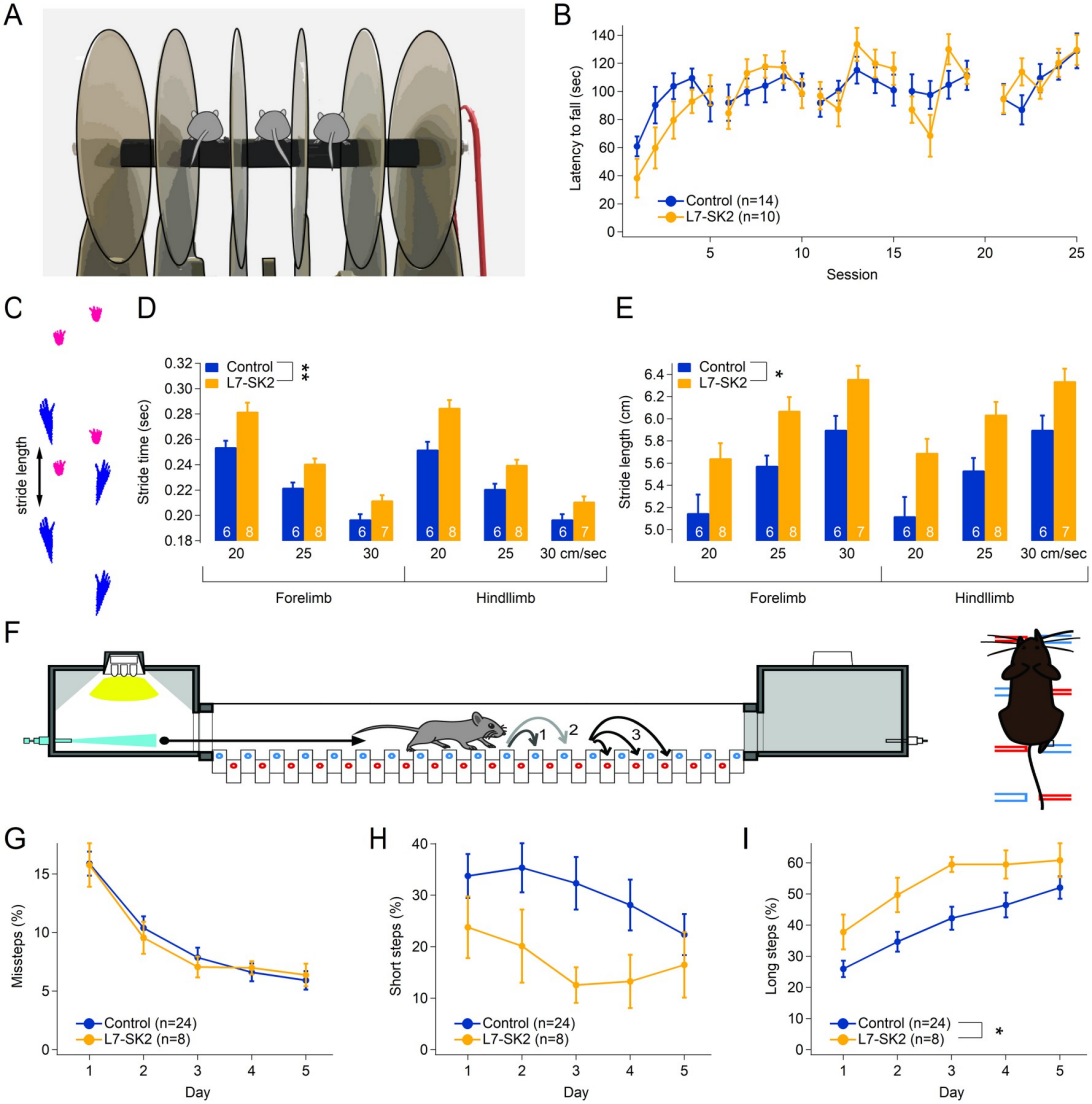
Gait patterns, but not overall walking performance, are altered in L7-SK2 mice. (A) Cartoon showing the layout of the rotarod apparatus. (B) Motor coordination on the rotarod was comparable in L7-SK2 and littermate WT control mice: the latency to fall was comparable, despite a nonsignificant trend for slower learning in L7-SK2 mice during the first 5 sessions corresponding to day 1. (C) Cartoon showing example paw prints (blue: hindlimbs, magenta: forelimbs) and stride length (arrow) as reconstructed from videos of a control mouse walking on a treadmill by the DigiGait software. (D–E) DigiGait analysis of mouse locomotion on a treadmill at fixed speed (20, 25, and 30 cm/s). The bar graphs show a significant increase in stride time (D) and a corresponding increase in stride length (E). (F) Cartoon depicting the design of the Erasmus Ladder, consisting of two shelter boxes on either side of a horizontal ladder that the mouse has to cross. Light and air puffs will nudge the mice to cross, typically using the upper (blue) bars and avoiding the lower (red) bars. Curved arrows indicate short (1), long (2), and lower steps (“missteps,” 3). (G) Over 5 days of testing, L7-SK2 mice make a similar number of missteps (lower rungs) as their WT controls. (H–I) L7-SK2 mice show a trend toward using fewer short steps (H) and more long steps (I), in line with the increased stride length observed in the DigiGait experiments. *p < 0.05; **p < 0.01. See also S2 and S3 Figs and S1 and S2 Tables. WT, wild type.

To test the role of SK2 in locomotion and overall motor performance, we first assessed, as a reference, the constitutive SK2-KO mice, which have a germline mutation similarly affecting the first two codons of *Kcnn2* with no cell-type specificity. This mutation was therefore expected to have a stronger impact on the behavioral phenotype than the more specific L7-SK2 mutation. Indeed, we observed that SK2-KO mice show visible tremors that are evident by eye starting from postnatal day 7–10 (P7–10) [48]. To obtain a quantitative description of the motor phenotype of these mice, we tested motor coordination on an accelerating rotarod (Fig 5A). Mice with an SK2-KO mutation showed very short latencies to fall or were unable to stay on the rod from the beginning. Although fall times significantly improved over training similar to those in control mice, they remained shorter than controls at all time points (S2A Fig; genotype effect: F[1, 8] = 20.40, *p* = 0.0020; session effect: F[24, 192] = 2.28, *p* = 0.0011; genotype × session interaction: F[24, 192] = 0.68, *p* = 0.86). We also analyzed locomotion in SK2-KO mice on a treadmill system (DigiGait; S2B–S2I Fig; S1 Table). Only 1 out of 11 SK2-KO mice could run at high speeds (30 cm/s), restricting our measurements to lower speeds (20 and 25 cm/s). We observed that SK2-KO mice had a statistically significant increase in gait parameters related to gait variability (stride-length coefficient of variance [CV], stance width CV, ataxia coefficient; S2E, S2G and S2H Fig) and of the paw angle specific for hind limbs (S2C Fig), providing a quantitative representation of their evident motor impairment. All the other major parameters were unaffected at both speeds (20 and 25 cm/s; stride time, stride width, S2B and S2F Fig). Only stride length showed a nonsignificant trend towards shorter values in SK2-KO mice, especially at 25 cm/s (S2D Fig).

We next tested L7-SK2 mice on the rotarod. In contrast to constitutive SK2-KO mice, L7-SK2 mice showed no significant impairment of rotarod performance (Fig 5A and 5B; genotype effect: F[1, 19] = 0.34, *p* = 0.56; genotype × session interaction: F[24, 456] = 1.10, *p* = 0.34), although on day 1, we recorded a nonsignificant trend for L7-SK2 toward shorter latency to fall time (genotype effect: F[1, 21] = 4.30, *p* = 0.051; genotype × session interaction: F[4, 84] = 0.82, *p* = 0.52). The lack of major performance deficits on the rotarod in L7-SK2 mice suggests that the motor control issues observed in SK2-KO mice resulted from alterations in neuronal cell types other than Purkinje cells. DigiGait analysis of locomotion performed at 20, 25, and 30 cm/s (Fig 5C–5E, S3 Fig, S2 Table) revealed that L7-SK2 mice show stance width variability and paw angles comparable to control littermates (S3D Fig). Also, the stance width was unaffected by the mutation as in SK2-KO mice (S3F Fig). Unexpectedly, in L7-SK2 mice we observed a significant improvement of stride-length CV (S3C Fig) and of the ataxia coefficient (S3A Fig). However, L7-SK2 mice showed significantly longer stride time (genotype effect: F[1, 11] = 11.72, *p* = 0.0057, Fig 5D) and length (genotype effect: F[1, 11] = 8.87, *p* = 0.013, Fig 5E) than their littermate controls at all treadmill speeds tested, parameters that were unaffected in SK2-KO mice.

Finally, we subjected L7-SK2 mice to the Erasmus Ladder, a horizontal series of pressure-sensitive rungs forcing the mice to precisely time and coordinate locomotion (Fig 5F, S3 Table). Because of the pattern of higher and lower rungs, mice are limited in their available step patterns, resulting predominantly in short steps (distance of 2 rungs), long steps (4 rungs) and lower steps (interpreted as missteps; these different types of step patterns are indicated by numbers 1, 2, and 3, respectively, in Fig 5F). L7-SK2 mice did not exhibit an increase in the percentage of missteps (Fig 5G), commonly seen in ataxia models. However, there was a change in step length, reflected by an increased percentage of long steps (genotype effect: F[1, 30] = 5.567, *p* = 0.025, Fig 5I) and a nonsignificant reduction of the percentage of short steps (genotype effect: F[1, 30] = 2.669, *p* = 0.113, Fig 5H). Together, these results suggest that although L7-SK2 mice have no major impairment in locomotion and do not show ataxia-like tremors, they display an abnormal locomotion pattern that results in taking significantly longer steps. Interestingly, we have seen a similar change in locomotion pattern—as well as impaired EBC—in a mouse model for the human 15q11-13 duplication, one of the most frequently observed genetic aberrations in autism, a brain developmental disorder that is associated with motor problems [51].

### Improved VOR gain adaptation in L7-SK2 mice

**Fig 6:**
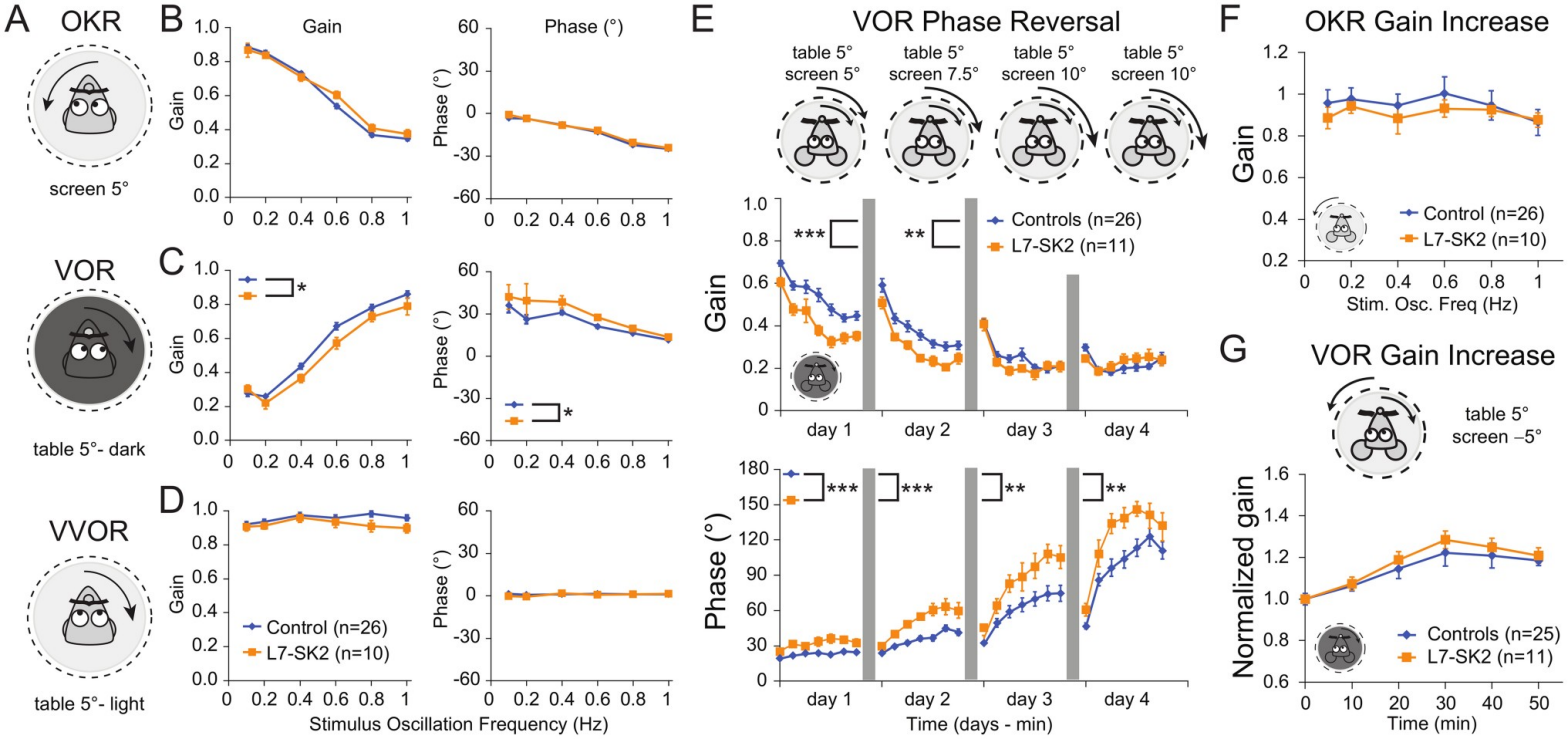
VOR baseline and adaptation. (A) Compensatory eye movements, aimed at minimizing retinal slip caused by visual and/or vestibular input, were used to test motor performance in L7-SK2 mice. L7-SK2 mice (*n* = 10) and controls (*n* = 26) were head-fixed on a table, and sinusoidal rotation (0.1–1.0 Hz) of the surrounding screen or the table evoked reflexive eye movements that were recorded by a table-mounted camera with IR lights. (B) Gain (measure of eye movement amplitude) and phase (measure of timing of the OKR evoked by rotation of the screen) did not differ between L7-SK2 and control mice. (C) The VOR, evoked by table rotation in the dark, was affected in L7-SK2 mice; both gain and phase were significantly impaired. (D) The combination of vestibular and visual input by rotation of the mouse in the light evoked the VVOR. With visual input, the VOR deficits in L7-SK2 were no longer present. (E) Next, mismatched visual and vestibular input was used to trigger adaptation of the eye movements in order to test motor-learning abilities in L7-SK2 mice. Training sessions (see cartoons) consisted of in-phase rotation of the vestibular and visual input with the same amplitude (5°, 0.6 Hz) on the first training day (day 1, also referred to as VOR gain decrease) and increasing amplitude of the visual input on days 2, 3, and 4. This training induced a reversal of VOR phase (from head to left = eyes to right to head to left = eyes to left), probed by VOR recordings in the dark (plotted data). L7-SK2 mice reversed their VOR phase faster than controls, with significantly higher phase values on all days. (F) Directly following the phase-reversal training, the OKR was probed again to determine OKR gain increase. Both L7-SK2 and control mice were able to increase their OKR gain compared to pretraining values in B. (G) Finally, the ability to perform VOR gain increase was also tested by subjecting mice to out-of-phase visual and vestibular input (5°, 0.6 Hz). Here, no effect of the mutation was observed in the ability to adapt the VOR gain. All data are mean ± SEM (note: for some data points, the error bars are obscured by the marker). *p < 0.05; **p < 0.01; ***p < 0.001. See also S4 Table. IR, infrared; OKR, optokinetic reflex; SEM, standard error of the mean; VOR, vestibulo-ocular reflex; VVOR, visually enhanced VOR.

In the rotarod test, L7-SK2 mice had similar learning curves as WT littermate controls in staying on the rotating rod, indicating similar motor adaptation of locomotion. To assess motor-learning phenotypes in L7-SK2 mice in a way that is more specific to the cerebellum, we examined the mice in an additional, well-established paradigm of cerebellum-dependent motor adaptation: their ability to perform and adapt their compensatory eye movements. We first tested basic performance parameters, including the amplitude (gain) and timing (phase), of the different compensatory eye movements: the optokinetic response (OKR; smooth eye movements driven by visual input, following oscillatory horizontal motion of a screen surrounding the animal), the VOR (smooth eye movements driven by vestibular input through rotation of the animal stage in the dark), and the visually enhanced VOR (VVOR; a combination of OKR and VOR by rotation of the animal in the light) at different oscillation frequencies (0.1–1 Hz; Fig 6A, S4 Table). The visually driven reflexes OKR and VVOR, but not the purely vestibular VOR, are most commonly attenuated in cerebellar mutants (e.g., [24, 52]). Interestingly, only the VOR was affected in L7-SK2 mice, not the OKR or VVOR (Fig 6B–6D, S4 Table). Next, we tested a phase-reversal VOR adaptation protocol, which is considered the type of VOR learning that is most sensitive to perturbations of the cerebellar circuit. VOR phase reversal aims to reverse the direction of the VOR (normally compensating the head rotation with an opposite eye movement to keep the image on the retina stable) using retinal slip caused by a screen rotation in the same direction (in phase) as the head rotation and with increasing amplitude each training day. This training ultimately resulted in an increase in the phase of the VOR in control mice (Fig 6E). L7-SK2 mice had lower VOR gains on the first 2 days of training and higher phase values on all days (Fig 6D, S4 Table), indicating that VOR phase reversal is faster in the absence of SK2 in Purkinje cells. At the end of the phase-reversal training, we retested the OKR. As a result of intense VOR training, OKR gains also increased (compare to Fig 6A). OKR gain increase appeared not to be altered in L7-SK2 mice compared to control littermates (Fig 6F, S4 Table). Finally, the same group was also tested for VOR gain increase, a short-term training paradigm aimed at increasing the gain of the VOR using out-of-phase visual and vestibular input. VOR gain increase was also not affected in L7-SK2 mice compared to control littermates (Fig 6G, S4 Table). Thus, L7-SK2 mice have a subtle phenotype with altered performance in compensatory eye movements and enhanced VOR phase-reversal learning, suggesting that the Purkinje-cell–specific loss of SK2 is sufficient to alter compensatory eye movements as well as their adaptation.

### EBC is impaired in L7-SK2 mice

**Fig 7:**
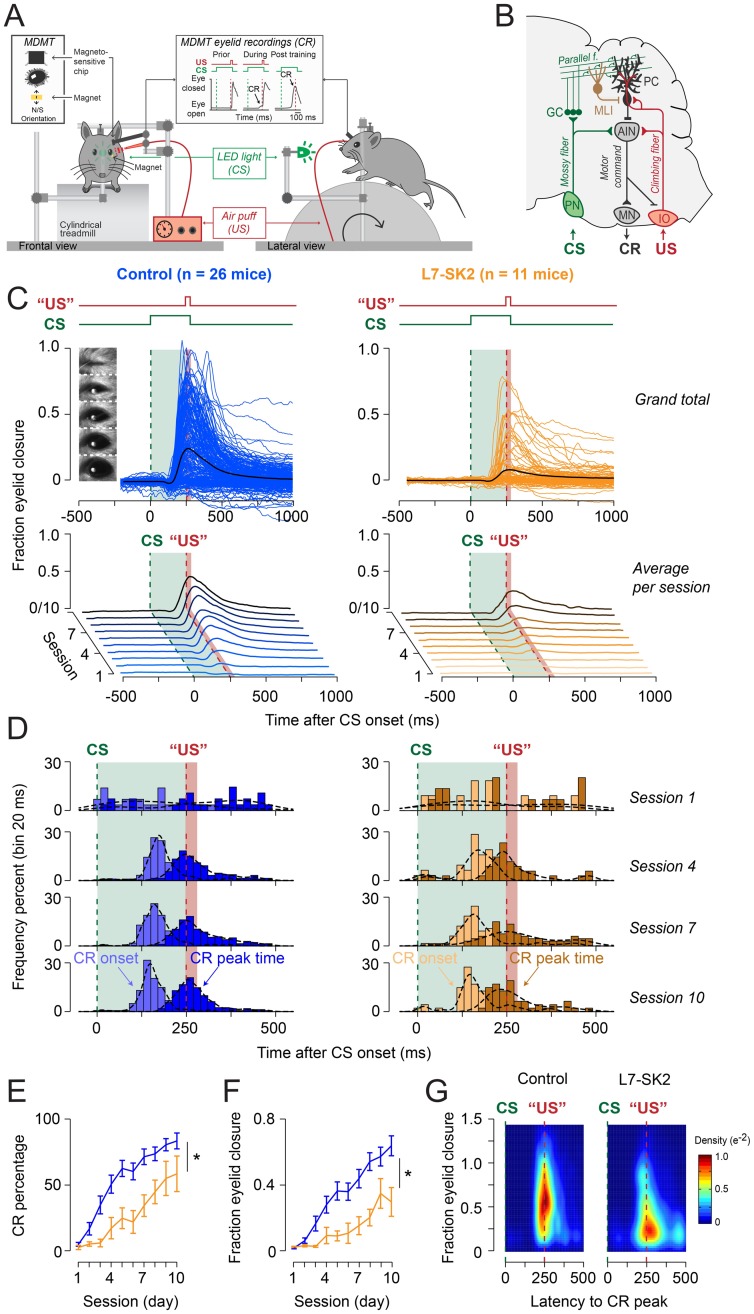
L7-SK2 mice show impaired EBC. (A) During EBC, mice are placed in a light- and sound-isolating chamber on a freely moving foam treadmill with their head fixed to a horizontal bar. Mice see a green LED light (CS), followed 250 ms later by a weak air puff on the eye (US). As a result of repeated CS–US pairings, mice will eventually learn to close their eye in response to the CS, which is called the CR. Eyelid movements were recorded with the MDMT. (B) During Pavlovian EBC, memory formation takes place in PCs of defined areas of the cerebellar cortex. These PCs receive inputs from the mossy fiber–PF system, which conveys sensory CS signals (green) and input from a single CF, which transmits the instructive US signal (red). During the conditioning process, these PCs acquire a well-timed suppression of their simple spike firing in response to the CS, thereby temporarily disinhibiting the cerebellar nuclei, which drives the overt eyeblink CR. (C) Upper panels: session-averaged (colored) eyeblink traces per mouse and grand total averaged (black) eyeblink traces during CS-only trials (total per panel 10 × 26 traces for control mice, 10 × 11 traces for L7-SK2 mice). Mouse eye video captures show eyelid closure ranging from 0 (fully open) to 1 (fully closed). Lower panels: waterfall plot showing the averaged eyeblink trace per group per session. (D) Peristimulus histogram plots with a Gaussian kernel density estimate (black dashed line) showing the distribution of CR onset (light filled bars) and CR peak time (dark filled bars) relative to CS and US onset in CS-only trials for sessions 1, 5, 10, and 15. In both groups, there is a clear development in CR onset and peak time: there are no clearly preferred times in the CS–US interval at the start of training (session 1), but during training, CR onset values are centered around 100–125 ms after CS onset, and CR peak times are located around the onset of the expected US. Although L7-SK2 plots look a little noisier, no significant difference could be established in any session for the mean value and/or standard deviation. Green dashed line is CS onset, red dashed line is US onset; light green and light red fill indicate CS and US duration, respectively. (E, F) L7-SK2 mice have lower CR percentage and FEC (or “amplitude of eyelid closure”) over the 10 acquisition sessions. (G) Two-dimensional density plot showing the fraction of eyelid closure relative to the latency to the CR peak over all sessions. The main impairment of L7-SK2 is in the spatial domain (i.e., amplitude or FEC) and less in the temporal domain (i.e., timing of CR peak). See also S5 Table. AIN, Anterior Interposed Nucleus; CF, climbing fiber; CR, conditioned response; CS, conditioned stimulus; EBC, eyeblink conditioning; FEC, fraction eyelid closure; GC, granule cell; IO, Inferior Olive; LED, light-emitting diode; MDMT, magnetic distance measurement technique; MLI, molecular layer interneuron; MN, motor neuron; NVE, NVE Corporation magnetic sensor; PC, Purkinje cell; PF, parallel fiber; PN, Pontine Nuclei; US, unconditioned stimulus.

SK2 channels control Purkinje cell excitability [43] and spike pauses [44]. In light of the role of simple spike suppression and spike pauses in the generation of motor output in general, and specifically the generation of CRs in EBC [28, 53, 54], we asked whether the Purkinje-cell–specific deletion of SK2 channels would result in EBC impairment. The training protocol used the association of a 280-ms light-emitting diode (LED) light flash as the CS, ending with a 30-ms air puff delivered to the mouse cornea as the US, which triggered the involuntary eyeblink response (UR, Fig 7A and 7B). We first looked at the group-averaged CR percentage and amplitude of the eyelid closures in response to the CS (i.e., fraction eyelid closure [FEC]). We found that L7-SK2 mice had a significantly lower overall CR percentage and amplitude of their eyelid closures (i.e., FEC) (CR percentage—group: F [1, 35] = 10.86, *p* = 0.002, FEC—group: F [1, 35] = 11.20, *p* = 0.002; all ANOVA on linear mixed-effect models [LMMs]) (Fig 7C, 7E and 7F; see also S5 Table). Next, we looked in more detail at the timing of the eyeblink CRs and found no significant effect for both latency to CR onset and latency to CR peak time between L7-SK2 and control mice (CR onset F [1, 35] = 0.50, *p* = 0.484; CR peak time: F [1, 35] = 0.14, *p* = 0.712, all ANOVA on LMMs). In both groups, latency to CR onset and CR peak time lacked a temporal preference in the first few sessions but clearly showed preference at the end of training. At this time, the average latencies to onset and to peak time of the CR were centered around 160 and 280 ms after CS onset, respectively. The latter corresponded to the time when the eyelid would be maximally closed in response to the US (CR onset F [1, 35] = 0.50, *p* = 0.484; CR peak time: F [1, 35] = 0.14, *p* = 0.712). Importantly, for analysis of CR timing, we corrected for FEC since the used criterion for CR detection (FEC > 0.1) easily picks up random small eyelid responses, which, as a matter of fact, do not have the proper timing like true cerebellar CRs. Since CR onset and CR peak time seemed slightly more “jittery” in L7-SK2 mice (Fig 7D), we also looked at the CV of these measures and found that both values trended to be slightly higher (CV CR onset F [1, 35] = 4.00, *p* = 0.053; CV CR peak time F [1, 35] = 3.36, *p* = 0.075). Together, our data show that L7-SK2 mice have impaired EBC in that they have less and smaller eyeblink CR, but the timing is relatively normal, at least for the one interval tested.

## Discussion

The key finding of this study is that a form of cerebellar motor learning that depends on simple spike suppression in Purkinje cells, delay EBC [28–30], is impaired in L7-SK2 knockout mice, which show intact bidirectional synaptic plasticity but altered intrinsic excitability and plasticity. A form of cerebellar learning that does not depend on simple spike suppression but has been linked to an increase in simple spike rate, gain-increase VOR adaptation [18, 45], showed a slight, yet significant, improvement in the L7-SK2 knockout mice. These findings demonstrate that cell-autonomous excitability regulation, separable from synaptic plasticity, is a central component in cerebellar learning, but with module-specific differences (see also [37, 45]).

VOR gain adaptation was not impaired, but instead more efficient, in L7-SK2 mice. A possible explanation is that Purkinje cells in the flocculus—responsible for VOR gain adaptation—are zebrin-positive and show significantly lower simple spike firing rates during spontaneous activity than zebrin-negative Purkinje cells (Fig 2) [35] while exhibiting an increase in simple spike activity during learning [18]. It is conceivable that a lack of SK2 channels allows Purkinje cells in the flocculus to operate at a slightly higher, more beneficial spike firing rate, resulting in an optimization of the modulation range. The mild alterations seen in the VOR gain adaptation paradigm as well as the trend for an increase in the spontaneous simple spike rate in the vestibulocerebellum (lobule X: from 47 Hz in WT mice to 58 Hz in L7-SK2 mice), suggest that excitability control may play a role here. In WT mice, this likely happens in combination with synaptic plasticity (see [24, 55, 56]). The change in VOR gain is in line with the idea that the firing rate of Purkinje cell populations can encode the timing and velocity of movements [57–60]. The enhanced increase in firing rate upon current injection in L7-SK2 Purkinje cells may also explain the longer steps observed in L7-SK2 mice according to recent work that shows that optogenetic inhibition of the cerebellar anterior interposed nucleus (which can be considered as an equivalent of an increase of the firing rate of Purkinje cells) is able to increase outward and upward velocity of forelimbs during reaching movements and causes overshooting of the target [61].

Delay EBC involves Purkinje cells near lobules V/HVI that, in their majority, are zebrin-negative [62]. These neurons operate at higher baseline simple spike firing rates [35], turning a pause in high-frequency firing into a robust and significant output signal when an acquired CR is expressed [45]. The observation that in L7-SK2 Purkinje cells from the zebrin-negative lobule III, we did not observe an increase in baseline simple spike rates shows that these neurons are not “locked in” to a permanent high-excitability state. In light of the critical requirement of simple spike suppression for EBC [28–31, 33], it is still possible that the observed alterations in excitability and the increase in evoked excitability, as well as the reduction in baseline spike firing, contribute to the robust EBC impairment in L7-SK2 mice. In this scenario, the enhanced excitability makes it harder to generate simple spike suppression after CS presentation. The lower baseline spike firing rate may contribute to the motor phenotype by preventing optimal activation of neurons in the cerebellar nuclei, which are more efficiently recruited when starting from higher firing rates [33, 63].

An observation from ex vivo studies of Purkinje cell properties after EBC learning that, at first inspection, seems contradictory to the postulated need for simple spike suppression is that AHP amplitudes are down-regulated and intrinsic plasticity is occluded [9–11], pointing toward enhanced, not reduced, excitability during EBC (see also [64]). It appears as if synaptic input activity during learning enhances excitability in Purkinje cells regardless of the type of learning, but that the EBC-specific memory trace—dominated by simple spike suppression—is made possible by additional cellular changes such as LTD and enhanced MLI activity [22].

What then is the function of Purkinje cell intrinsic plasticity in EBC motor learning? Is it even possible to conclude that the phenotypic observations in L7-SK2 mice at least partially result from prevention of intrinsic plasticity rather than differences in excitability itself? It is important to note that intrinsic plasticity is selectively impaired in L7-SK2 mice, while its synaptic counterparts, LTD and LTP, stay intact. To be sure, the absence of intrinsic plasticity is not absolute. As we have previously reported [44], a small late-onset component of excitability change can be observed under strong activation conditions in SK2 knockout mice. However, because the amplitude of this effect is <15% and it occurs only about 10 min after tetanization, this remaining plasticity component is likely not sufficient to support EBC motor learning. Thus, it is justified to consider L7-SK2 Purkinje cells as incapable of supporting physiologically relevant intrinsic plasticity. A comparison with cellular and behavioral phenotypes in a second mouse model with a lack of SK2-mediated intrinsic plasticity in Purkinje cells, L7-PP2B mice, supports the hypothesis that indeed, SK2-mediated intrinsic plasticity may play a critical role in EBC motor learning. In L7-PP2B mice, calcineurin (PP2B) is absent from Purkinje cells, causing not only a blockade of intrinsic plasticity (and LTP) but also a decrease in membrane excitability [20, 24]. In L7-PP2B mice, CR acquisition in the EBC task was similarly affected as in L7-SK2 mice, with an opposite change in basic excitability [24] (see also [65, 66] for similar observations in L7-SHANK-2 and L7-STIM1 mice, respectively). Together with our observations from L7-SK2 mice presented here, these results suggest that the ability to up-regulate Purkinje cell excitability in an experience-dependent way may provide a requirement for the proper and complete execution of EBC motor learning.

This is a surprising result in light of the need for simple spike suppression to enable eyelid closure. Why is enhanced excitability needed if the memory trace itself carves out a period of pause in spike firing to enable eyelid closure? One possibility is contrast enhancement. Simple spike suppression will be a stronger effector in eyelid closure in an environment of high excitability and optimal firing rates outside the memory trace itself. Enhanced excitability may also keep Purkinje cells ready to initiate alternative, possibly overriding, memory patterns and/or to properly terminate a memory trace to avoid the intermixing of more than one memory trace [67]. In addition, it is conceivable that enhanced excitability of Purkinje cells also plays a role in inhibiting the antagonistic muscles that are responsible for opening the eyelid; indeed, these Purkinje cells may increase their activity during eyelid closure [28], while their targets in the cerebellar nuclei that drive the opening reduce their activity [33]. Finally, it is conceivable that intrinsic plasticity amplifies PF and/or CF inputs in a way that is related to the memory trace itself. SK2 plasticity shows functional proximity to LTP at PF synapses, ensuring that potentiated synapses can impact Purkinje cell output [68]. This effect might be important to stabilize/potentiate CS-related, as well as non-CS–related, PF inputs that do not contribute to the error signal but instead promote beneficial motor programs. Indeed, synaptic and intrinsic plasticity co-occur in the cerebellar cortex and are likely to complement each other [43, 69–71].

An alternative scenario relates to the puzzling observation that during conditioning, complex spikes emerge in response to CS application [28, 30]. The driving mechanism for these CS complex spikes is currently not understood, and neither is their function, but we know that SK2 plasticity amplifies complex spikes by enhancing both the dendritic responses as well as the spikelet number [11, 72]. Such amplification might affect both CS- and US-evoked complex spikes, which appear to show different spikelet configurations [73], and may facilitate PF-LTD [74, 75]. Because the complex spike pause is not reduced in SK2-KO mice under basic conditions [44], the synaptically driven alteration in the simple spike pattern probably does not add to the list of excitability parameters that are altered in the absence of SK2 channels. The plasticity of pause duration—a consequence of intrinsic plasticity—is also absent from these mice [44]. This plasticity results in a shortening of the complex spike pause but does not contribute in general to the simple spike suppression itself that is a hallmark of conditioned Purkinje cells. However, the shortening of this type of pause further supports the notion that SK2 plasticity may enhance the responsiveness of Purkinje cells to synaptic input and possibly in particular those that are related to the CS.

In summary, our study shows that SK2 channels, likely through their participation in the postburst AHP, are needed to adjust Purkinje cell membrane excitability to preferred levels in a motor-learning context. There are three main consequences of the absence of SK2 expression that need to be considered: a) a change in spontaneous simple spike activity in specific parts of the cerebellar cortex, b) a shift in excitability, and c) the absence of an intrinsic plasticity component that rests on SK2 down-regulation but is occluded in these mice. All three cellular phenotypes have in common that they result from the inability of L7-SK2 Purkinje cells to modulate their membrane excitability appropriately. It is this inability to adjust spike patterns that eliminates the difference in baseline and evoked spiking between Purkinje cells from zebrin-positive and zebrin-negative lobules and that thus removes the unique conditions that facilitate simple spike suppression during EBC learning. These findings—together with the observation from previous ex vivo studies that the Purkinje cell AHP is reduced after EBC learning, an effect that probably enhances responsiveness of those Purkinje cells that were activated during conditioning—allow for the identification of an intrinsic plasticity component as part of a wider network of plasticity mechanisms and places [19, 20] that is essential for a proper and complete execution of a specific type of behavioral learning.

## Materials and methods

### Ethics statement

All animal experiments were approved and carried out in accordance with the regulations and guidelines for the care and use of experimental animals at the Institutional Animal Care and Use Committee of the University of Chicago (according to National Institute of Health guidelines; IACUC 72000) or at the Institutional Animal Welfare Committee of the Erasmus Medical Center (according to the Dutch transposition of the European directive on scientific animal experimentation 2010/63/EU; AVD101002015273).

### Animals

All mouse strains were maintained on a C57BL/6J background (The Jackson Laboratory, Bar Harbor, ME, USA) by periodic backcrossing with C57BL/6J mice. Homozygous pups for SK2-KO mutation were obtained from couples of heterozygous mice and could be consistently recognized from P7–10 by their tremors. Feeding competition was limited by reducing the litters to 3–4 pups. Growth was assisted with a high-fat diet provided to breeding pairs and by delayed weaning and nutritional gel after weaning. Littermates were group-housed in standard conditions (12-h light/dark cycles and water and food ad libitum) with nesting material. Both male and female mice were included. For eye movement recordings, Erasmus Ladder, and EBC experiments, 12- to 40-week–old male and female mice were used, individually housed with food ad libitum and 12:12 light/dark cycles.

### Generation of L7-SK2 mice

Mice with a Purkinje cell-specific null mutation for *Kcnn2* locus encoding for SK2 protein (“L7-SK2” mutant) were obtained with the support of the Transgenic Mouse Facility at the University of Chicago (Chicago, IL, USA) using the CRE-Lox-system, with LoxP sites flanking the first two coding exons and comprising the initiation of translation codons of both the “short” [48] and “long” isoform of the gene (“*Kcnn2L*”; [76]) in order to disrupt both isoforms in an exclusively CRE-dependent manner. Both isoforms are also absent in the constitutive SK2-KO [48], which was obtained with an upstream LoxP site placed between the two start codons [48, 76], disrupting the long isoform in a CRE-independent manner. The genomic DNA was obtained from a male C57BL/6J genomic library. To generate the new L7-SK2 mutant, the downstream LoxP site was inserted together with a downstream Neo cassette flanked by FRT sites for selection of recombinant clones. Gene targeting was achieved by electroporation and homologous recombination with in C57BL/6N-tac mouse embryonic stem cells (PRX-B6N #1; Primogenix, St. Louis, MO, USA), tested by PCR for insertion of the Neo cassette (primers 5′ to 3′: Fw, TCCCCGCGGACTTCTTCAGGCTGGTACACTTCTAC; Rev, CCGCTCGAGACTCTCAGTGGTGCAGGACTCTTGGTG) and confirmed by Southern blot (restriction enzyme: BglII; primers for probe generation, 5′ to 3′: Fw, TAGTTGGTAGGCTAGTGTCC; Rev, AAATTACTCATCAGCACTCA).

Male chimeric mice, obtained by injecting a positive clone in blastocytes, were crossed with C57BL/6J females. The Neo cassette (flanked by FRT sites) was excised by crossing with FLP-expressing mice (JAX#005703). The Flp cassette was eliminated by further crossing the mice on C57BL/6J background (The Jackson Laboratory), on which the strain was maintained (“SK2-floxed”; proposed formal name according to The Jackson Laboratory Mouse Strain nomenclature: B6-*Kcnn2*^*tm3Crwh*^). A mouse strain expressing the CRE recombinase under the PC-specific promoter Pcp2/L7 (JAX#010536, [47]) was used to obtain Purkinje cell-specific SK2-KO mice (“L7-SK2” strain). Mice homozygous for SK2-loxP (SK2^loxP/loxP^) were crossed with mice heterozygous for both SK2-loxP and the L7-Cre transgene (SK2^+/loxP^ L7-Cre^+^).

Mice of the following genotypes were used for the experiments: “L7-SK2” mice (SK2^loxP/loxP^ L7-Cre^+^) and control littermates (SK2^loxP/loxP^ L7-Cre^−^; SK2^+/+^ L7-Cre^+^; SK2^+/+^ L7-Cre^−^).

### Mouse genotyping

The genotypes of the offspring of SK2-KO strain and of the final L7-SK2 strain were analyzed on tail genomic DNA by PCR or by a commercial genotyping service (Transnetyx). The following are the primers used for the L7-SK2 strain to detect the downstream LoxP site (5′ to 3′): Fw, TCCCCGCGGACTTCTTCAGGCTGGTACACTTCTAC, Rev, CCGCTCGAGACTCTCAGTGGTGCAGGACTCTTGGTG to amplify an approximately 670-bp WT amplicon or an approximately 740-bp LoxP^+^ amplicon; the primers for the Cre cassette (5′ to 3′) are Cre-Fw, GCGGTCTGGCAGTAAAAACTATC, Cre-Rev, GTGAAACAGCATTGCTGTCACTT, control-Fw, CTAGGCCACAGAATTGAAAGATCT, control-Rev, GTAGGTGGAAATTCTAGCATCATCC, to amplify an approximately 100-bp Cre^+^ amplicon and an approximately 320-bp internal control amplicon; the primers used to control for a constitutive CRE-dependent excision (5′ to 3′) are Fw, TCCCCGCGGGTCTGCGCCGTAGCGCTCTCCCGTAG, Rev: CCGCTCGAGACTCTCAGTGGTGCAGGACTCTTGGTG to amplify an approximately 700-bp SK2^−^ amplicon (in case of inherited germline deletion). The general PCR program used was as follows: denaturation at 95 °C for 60 s; 31 cycles of denaturation at 98 °C for 10 s and annealing/elongation at 68 °C for 2 min; final single elongation step at 68 °C for 2 min; enzyme: Takara Ex Taq Hot Start Version (RR006A; Takara Bio USA, Mountain View, CA, USA). The upstream LoxP site could be checked for further control if needed with the following primers (5′ to 3′): Fw, TCCCCGCGGGTCTGCGCCGTAGCGCTCTCCCGTAG; Rev, GTGCATCTGGTACCACGGTTGGGAAGAC.

### Histology

#### Immunohistochemistry

For calbindin immunostaining, mice (6–7 months old and 9–10 months old, respectively, for the SK2-KO and L7-SK2 mouse strains) were deeply anesthetized by ketamine/xylazine and intracardially perfused with ice-cold 4% paraformaldehyde. Brains were postfixed for 2–16 h and equilibrated with 30% sucrose overnight. Sagittal sections (50 μm) were permeabilized in PBS with Triton-X 0.25% (Tx-PBS) and treated as follows in Tx-PBS based solutions: they were preincubated with 10% normal donkey serum solution (Jackson Immunoresearch USA, West Grove, PA, USA) for 1 h at room temperature (RT) and incubated with mouse anti-Cb (1:1,000; Swant, Marly, Switzerland; code 300) overnight at 4 °C, then incubated with secondary antibody (Alexa 488-conjugated anti-mouse, 1:200; Jackson Immunoresearch) for 2 h at RT and rinsed. For the quantification of Purkinje cell density, images were acquired with a Zeiss LSM 5 Exciter confocal microscope (10× objective, 0.5× digital zoom for the SK2-KO strain; Carl Zeiss, Oberkochen, Germany) or a Pannoramic SCAN II by 3DHISTECH-Perkin (20× objective for the L7-SK2 strain; 3DHISTECH, Budapest, Hungary). Exported images were analyzed by NIH ImageJ software. Calbindin-positive Purkinje cells were counted in random fields (5–10/animal), and their number was normalized by the length of the Purkinje cell PC layer section that was analyzed and averaged within each animal.

#### In situ hybridization (RNAscope)

The expression of *Kcnn2* RNA was tested automatically with ACDBio RNAscope 2.5 assay in situ hybridization system (Advanced Cell Diagnostics, Newark, CA, USA) on Leica Bond RX (Leica, Biosystems USA, Buffalo Grove, IL, USA) at the histology core facility (Human Tissue Resource Center) at the University of Chicago. Briefly, brains (3-month–old L7-SK2 mice or littermates, 9-month–old SK2-KO mice) were fixed for 28–31 h by immersion in 10% formalin buffer immediately after the mice were killed, then transferred in 70% ethanol and paraffin-embedded. Sections (5–10 μm) were treated according to the protocol and treated with the specific probe (LS 2.5 Probe Mm-Kcnn2, Cat. no. 427978; Advanced Cell Diagnostics, Inc., Newark, CA, USA), the 2.5 LS Duplex Reagent Kit (322440, Advanced Cell Diagnostics), and stained in DAB chromogenic brown color (pretreatment/digestion: 15 min, 95 °C).

#### Golgi staining

Golgi staining was performed using an FD Rapid Golgi Stain Kit (FD NeuroTechnologies, Columbia, MD, USA). Stained slices (135 μm) were obtained from 2.5- to 4-month–old mice, imaged with a Zeiss AxioCam MRm camera and a 310 IR-Achroplan objective, and mounted on a Zeiss Axioscope 2FS microscope (Carl Zeiss MicroImaging). Images were analyzed by NIH ImageJ software and NeuronJ plug-in software as previously described [44] to measure the total length of the dendritic arbor and the length of the primary dendrite. Sholl analysis of dendritic arbors was performed by the Sholl analysis plug-in in ImageJ, counting the number of intersections per radius step with increasing distance from the soma (5-μm starting radius and steps).

### Electrophysiology

Sagittal slices of the cerebellar vermis (200 μm) were prepared from P25-36 C57BL/6J mice after isoflurane anesthesia and decapitation. P21-136 mice were used for the recordings, in which we tested for the elimination of surplus climbing fibers. Slices were cut with a Leica vibratome (VT1000S) and ceramic blades in ice-cold artificial cerebrospinal fluid (ACSF) containing the following: 124 mM NaCl, 5 mM KCl, 1.25 mM Na_2_HPO_4_, 2 mM MgSO_4_, 2 mM CaCl_2_, 26 mM NaHCO_3_, and 10 mM D-glucose, bubbled with 95% O_2_/5% CO_2_. The slices were kept at RT in ACSF for at least 1 h, then transferred to a submerged recording chamber superfused with ACSF supplemented with picrotoxin (100 μM) to block GABA_A_ receptors at RT (for recordings of synaptic currents) or near-physiological temperature (31 °C–34 °C, for recordings of spike firing). Whole-cell patch-clamp recordings were performed under visual control with differential interference contrast optics combined with near-infrared light illumination (IR-DIC) using a Zeiss AxioCam MRm camera and a 340 IR-Achroplan objective, mounted on a Zeiss Axioscope 2FS microscope (Carl Zeiss MicroImaging). For whole-cell patch-clamp recordings (except for CF recordings) and cell-attached recordings, patch pipettes (whole cell: 2.5–4.5 MΩ; cell attached: 4–6 MΩ) were filled with internal saline containing the following: 9 mM KCl, 10 mM KOH, 120 mM K-gluconate, 3.48 mM MgCl_2_, 10 mM HEPES, 4 mM NaCl, 4 mM Na_2_ATP, 0.4 mM Na_3_GTP, and 17.5 mM sucrose, with pH adjusted to 7.25–7.35 with KOH and osmolarity adjusted to 295–305 mmol/kg with sucrose. For cell-attached recordings, the NMDA receptor antagonist D-AP5 (50 μM) and the AMPA receptor antagonist NBQX (10 μM) were added to the bath. For CF recordings (Fig 2C), the internal solution was composed of 100 mM CsMeSO4, 50 mM CsCl, 1 mM MgCl2, 0.2 mM EGTA, 10 mM HEPES, 2 mM Na2ATP, 0.3 mM Na3GTP, and 4 mM QX-314 (pH 7.25–7.35). For determining the number of CF inputs by testing different stimulus intensities at varying locations (Fig 2D), the internal solution was composed of 60 mM CsCl, 10 mM Cs D-Gluconate, 20 mM TEA-Cl, 20 mM BAPTA, 4 mM MgCl_2_, 4 mM Na_2_ATP, 0.4 mM Na_3_GTP, and 30 mM HEPES (pH 7.34). Data were acquired with an EPC-10 amplifier and Patchmaster software (HEKA Electronik, Lambrecht/Pfalz, Germany) and digitized at 20 kHz (voltage clamp) or 10 kHz (current clamp). Membrane potential was corrected for liquid junction potential (7.5 mV for CF-EPSC recording and 11.7 mV for other recordings). Bridge compensation (80%) was applied in current-clamp mode. Cells were held at −80 mV to record PF-EPSCs, at 0 mV to −20mV for CF-EPSCs, and at −66 to −73 mV in current-clamp mode (to prevent spontaneous spike activity). For synaptic stimulation, a glass pipette filled with ACSF was placed in the granular or molecular layer for PF or CF activation, respectively, and a current pulse was applied (0.2–0.5 ms). To assess PF and CF synaptic transmission, EPSCs were recorded every 20 s at increasing stimulation intensity (for PF) or at increasing interstimulus intervals (to test pair-pulse ratio), averaging 4–6 tests per point. For PF plasticity experiments, the stimulation intensity was set for each cell to evoke a 150–300 pA PF-EPSC, then kept constant throughout the recording. The applied LTD and LTP induction protocols consisted of a single PF or paired single PF + CF stimulation at 1 Hz for 5 min in current-clamp mode. Intrinsic excitability was assessed by counting the action potentials that were evoked by depolarizing currents of increasing amplitude. For intrinsic plasticity, Purkinje cell intrinsic excitability was monitored during the test periods by injection of brief (500-ms) depolarizing current pulses (150–600 pA every 20 s) adjusted for each cell to evoke approximately 15–30 spikes and kept constant throughout the experiment. The induction protocol consisted of the injection of depolarizing currents (500–900 pA, 100 ms) at 5 Hz for 8 s. For plasticity experiments, all values were averaged over time (3 successive responses recorded at 20 s intervals) and normalized over a 5-min baseline. In voltage-clamp recordings, the input resistance (Ri) and series resistance (Rs) were measured by applying a hyperpolarization step (10 mV), while in current-clamp recordings, Ri was measured by injection of hyperpolarizing test currents (100 pA). Cells with a change higher than 15% in voltage clamp for Ri and Rs or in current clamp for Ri or holding potential (Vh) were excluded from the analysis. Drugs were purchased from Sigma-Aldrich (St. Louis, MO, USA) or Tocris (Bristol, UK). Data were analyzed with Patchmaster (HEKA Elektronik), Igor (Wavemetrics, Portland, OR, USA), and Excel (Microsoft, Redmond, WA, USA). Artifact spikes were deleted from shown EPSC traces for visualization purposes.

### Motor behavior

#### Rotarod

The accelerating rotarod test was performed using a computer-controlled rotarod apparatus (Rotamex-5; Columbus Instruments, Columbus, OH, USA) with a rod (7-cm diameter, 4 lanes) that was set to accelerate from 4 to 40 rpm over 300 s, and time to fall was recorded (start speed = 4 rpm; acceleration steps: 0.1 rpm/0.8 s). Mice (7.5–18 months old or 6–14 weeks old, respectively, for SK2-KO or L7-SK2 mouse strains) received 5 consecutive trials per session at 1 session per day for 5 days with at least 30–60 s intertrial intervals (ITIs). The latency to falling time was recorded automatically by photosensors, and the mean time on the rod on each day was analyzed.

#### DigiGait

Mouse gait was analyzed using the DigiGait imaging system (version 4.0.0; Mouse Specifics, Framingham, MA, USA). Mice (4- to 13-week–old SK2-KO or WT and 4- to 5-week–old L7-SK2 or WT mice) were placed on a motorized transparent treadmill within a plexiglass compartment. The belt was turned on at an initial speed of 5–10 cm/s. The speed was gradually increased from 5–10 cm/s to a constant test speed of 20–30 cm/s. Digital videos of paw placement were acquired using a camera mounted beneath the treadmill. For each mouse, 2–5 clips of 2.5–5 s each were selected on the basis of continuous movement and absence of gait pauses. Video acquisitions were repeated until a sufficient number of clips were obtained for each speed. The videos were analyzed using DigiGait software (version 14), which automatically processed the video images to calculate gait parameters. Visual inspection of the analysis and manual corrections were systematically performed for each clip. Parameters were exported and further analyzed in Excel (Microsoft), calculating mean values per animal and per speed for fore- and hindpaws separately, pooling together left and right paws.

#### Erasmus Ladder

The Erasmus Ladder consists of a horizontal ladder between two shelter boxes, each equipped with an LED spotlight in the roof and two pressurized air outlets in the back. Sensory stimuli (light and air) serve to control the moment of departure of the mice. The ladder itself has 37 rungs on each side, and each rung can be displaced vertically following a command from the control system. Even-numbered rungs on one side and odd-numbered rungs on the other were elevated by 6 mm, thereby creating a left/right alternating pattern. All rungs are equipped with custom-made pressure sensors that are continuously monitored. The setup is controlled by software written in LabView (National Instruments, Austin, TX, USA) that operates with a fixed cycle of 2 ms. For the current study, we followed a paradigm similar to that of a previous study [77]. Each mouse had to perform one daily session for 5 days. Each daily session consisted of 72 trials, during which the mouse had to walk back and forth between the two shelter boxes. During these sessions, we assessed locomotion parameters in all mice as they crossed the rungs between the boxes (for details, see [78, 79]). Steps were recorded as touches on the rungs; to prevent false positives, we took into account only touches that lasted >30 ms. To reduce the potential impact of a putative bias due to the air and/or light stimuli in the shelter box, the first and last steps of each trial (i.e., stepping out of and into the shelter boxes) were omitted from analyses. To avoid detecting hind limb touches as backward steps, we accepted only sequences of two or more consecutive backward steps as true backward movements. The analyses of forward steps revealed that mice usually step from one elevated rung to the next, skipping the lower rung (i.e., step length = 2), or to the consecutive elevated rung, skipping three rungs (i.e., step length = 4). Hence, we considered steps with a step length equal to 2 or 4 to be “short steps” or “long steps,” respectively. Stepping on a lower rung was labeled here as “lower steps” or “missteps.” Other step types occurred less frequently and are hence not presented here. Step time was defined as the time that elapsed between the onsets of two consecutive touches of the same limb.

Data collected from the Erasmus Ladder were stored in a relational database (MySQL, Oracle, Redwood Shores, CA, USA) and then processed offline using custom-written software in LabView and Python (code available on request) (Python Software Foundation, Beaverton, OR, USA). Statistical analyses were conducted in SPSS (SPSS Inc., Armonk, NY, USA). All data were subsequently analyzed with repeated-measures ANOVA. Data sets were analyzed with the investigator blinded to the genotype of the animals.

#### Compensatory eye movements

Mice were surgically prepared for head-restrained recordings of compensatory eye movements. A small construct (“pedestal”) was attached to the skull after shaving and opening the skin overlaying it, using Optibond primer and adhesive (Kerr, Bioggio, Switzerland) and under isoflurane anesthesia in O_2_ (induction with 4% and maintained at 1.5% concentration). Mice were administered xylocaine and an injection with bupivacaine hydrochloride (2.5 mg/ml, bupivacaine actavis) to locally block sensation. The pedestal consisted of a brass holder (7 × 4 mm base plate) with a neodymium magnet (4 × 4 × 2 mm) and a screw hole for fixation.

After a recovery period of at least 3 days, mice were placed in a mouse holder, using the magnet and a screw to “catch” and fix the pedestal to a custom-made restrainer, and the mouse was placed with the head in the center on a turntable (diameter 60 cm) in the experimental setup. A round screen with a random dotted pattern (“drum,” diameter 63 cm) surrounded the mouse during the experiment. The recording camera was calibrated by moving the camera left–right by 20° peak to peak at different light levels (see below). Compensatory eye movement performance was examined by recording the OKR, VOR, and VVOR using a sinusoidal rotation of the drum in light (OKR), rotation of the table in the dark (VOR), or rotation of the table (VVOR) in the light [80]. These reflexes were evoked by rotating the table and/or drum at 0.1 to 1 Hz (20 to 8 cycles, each recorded twice) with a fixed 5° amplitude. In order to evaluate motor learning, a mismatch between visual and vestibular input was used to adapt the VOR. The ability to perform VOR phase reversal was tested using a 4-day paradigm, consisting of six 5-minute training sessions every day with VOR recordings before, between, and after the training sessions. On the first day during training, the visual and vestibular stimuli rotated in phase at 0.6 Hz and at the same amplitude, inducing a decrease of gain. On the subsequent days, the drum amplitude was increased relative to the table and induced the so-called phase reversal of the VOR, resulting in a compensatory eye movement in the same direction as the head rotation instead of the normal compensatory opposite direction (all days vestibular 5° rotation, visual day 1: 5°; day 2, 7.5°; days 3–4, 10°). Between recording sessions, mice were kept in the dark to avoid unlearning of the adapted responses. On the last day, the session was finished by again testing OKR at all frequencies (0.1–1.0 Hz) with 5° amplitude to evaluate OKR gain increase as a result of the VOR phase-reversal training paradigm. In a separate experiment, VOR gain increase was tested by rotating the visual and vestibular stimuli out of phase (at the same amplitude and at 0.6 Hz) for 5 training sessions of 10 min each.

Eye movements were recorded with a video-based eye-tracking system (hard- and software, ETL-200; ISCAN systems, Burlington, MA, USA). Recording were always taken from the left eye. The eye was illuminated during the experiments using two table-fixed infrared emitters (output 600 mW, dispersion angle 7°, peak wavelength 880 nm) and a third emitter that was mounted to the camera, aligned horizontally with the optical axis of the camera, which produced the tracked corneal reflection. Pupil size and corrected (with corneal reflection) vertical and horizontal pupil position were determined by the ISCAN system, filtered (CyberAmp; Molecular Devices, San Jose, CA, USA), digitized (CED, Cambridge, UK) and stored for offline analysis. All eye movement signals were calibrated [81], differentiated to obtain velocity signals, and high-pass–filtered to eliminate fast phases, and then cycles were averaged. Gain—the ratio of eye movement amplitude to stimulus amplitude—and phase values—time difference between eye and stimulus expressed in degrees—of eye movements were calculated using custom-made MATLAB routines (The MathWorks, Natick, MA, USA).

#### EBC

Surgery: Mice were anesthetized with an isoflurane/oxygen mixture (5% for induction, 1.5–2% for maintenance), and body temperature was kept constant at 37° Celsius. Eyes were protected against drying using an eye lubricant (Duratears; Alcon, Geneva, Switzerland). After a local scalp injection of bupivacaine hydrochloride (2.5 mg/ml, bupivacaine actavis), we made a sagittal scalp incision of 2–3 cm length. Next, we carefully removed the exposed periosteum and roughened the surface of the skull using an etchant gel (Kerr). After this, a small messing block (1.0 × 0.4 × 0.3 mm) with 1 screw thread and 2 additional pinholes was placed on the skull using Optibond primer and adhesive (Kerr) and Charisma (Heraeus Kulzer, Armonk, NY, USA). The surgical placement of this so-called pedestal allowed for head fixation during the EBC experiments [22].

Behavioral training: All behavioral experiments were conducted using head-fixed mice that were placed on top of a cylindrical treadmill on which they were allowed to walk freely (Fig 7A) The treadmill consisted of a foam roller (diameter ±15 cm, width ±12 cm), with a horizontal metal rod through the axis that was connected with a ball-bearing construction to two solid vertical metal poles. A horizontal messing bar was fixated to the same vertical poles at 3–5 cm above the treadmill. Mice were head-fixed to this bar using 1 screw and 2 pins, thereby ensuring perfect head fixation (for further details, see [81]). National Instruments (NI-PXI) equipment was used to control experimental parameters and to acquire the eyelid position signal. Eyelid movements were recorded with the magnetic distance measurement technique (MDMT), which makes use of an NVE giant magnetoresistance (GMR) magnetometer (NVE Corporation, Eden Prairie, MN, USA), positioned above the left upper eyelid, that measures movements of a minuscule magnet (1.5 × 0.7 × 0.5 mm) that is placed on the left lower eyelid of the animal with superglue (cyanoacrylate). This way, MDMT allows high spatiotemporal detection of eyelid kinematics (for further details, see [82]). The CS was a green LED light (CS duration 280 ms, LED diameter 5 mm) placed 10 cm in front of the mouse’s head. Because we performed our experiments in almost complete darkness, this small LED light was a salient stimulus that could be easily detected by both eyes. The US consisted of a weak air puff applied to the eye (30 psi, 30-ms duration), which was controlled by an ASI MPPI-3 pressure injector (Applied Scientific Instrumentation, Eugene, OR, USA) and delivered via a 27.5-gauge needle that was perpendicularly positioned at 0.5–1 cm from the center of the left cornea. The training consisted of 3 daily habituation sessions, 1 baseline measurement, and 10 daily acquisition sessions [83]. During the habituation sessions, mice were placed in the setup for 30–45 minutes, during which the air puff needle (for US delivery) and green LED (for CS delivery) were positioned properly, but no stimuli were presented. On the day of acquisition session 1, each animal first received 20 CS-only trials as a baseline measure to establish that the CS did not elicit any reflexive eyelid closure. During each daily acquisition session, every animal received in total 200 paired CS–US trials, 20 US-only trials, and 20 CS-only trials. These trials were presented in 20 blocks; each block consisted of 1 US-only trial, 10 paired CS–US trials, and 1 CS-only trial. Trials within the block were randomly distributed, but the CS-only trial was always preceded by at least two paired CS–US trials. The interval between the onset of CS and that of US was set at 250 ms. Because of an inherent 14-ms delay in the delivery of the air puff, we triggered the air puff at 236 ms after CS onset so that it would hit the cornea exactly at 250 ms after CS onset. The ITI was set according to the following constraints: at least 10 s had to elapse, the eyelid had to be open below a predetermined threshold of 50% of a full eyelid closure, and eyelid position had to be stable for at least 2 seconds for a trial to begin. During all training sessions, the experimenter carefully inspected threshold and stability parameters and adjusted them if necessary. All experiments were performed at approximately the same time of day by the same experimenter.

Data analysis: Individual eyeblink traces were analyzed automatically with custom computer software (LabVIEW or MATLAB). First, 2,000-ms eyeblink traces were imported and filtered in forward and reverse direction with a low-pass Butterworth filter using a cutoff frequency at 50 Hz. Second, trials with significant activity in the 500 ms pre-CS period (>7 × interquartile range) were regarded as invalid for further analysis. Third, trials were normalized by aligning the 500 ms pre-CS baselines and calibrating the signal so that the size of a full blink was 1 (FEC). Fourth, in valid normalized trials, all eyelid movements larger than 0.1 and with a latency to CR onset between 50–250 ms and a latency to CR peak of 100–250 ms (both relative to CS onset) were considered as CRs. For CS-only trials in the probe session, we used the exact same criteria, except that the latency to CR peak time was set at 100–500 ms after CS onset. Additionally, we determined for each individual trial the following parameters: (1) maximum eyelid closure (= FEC) in the CS–US interval, (2) CR amplitude, (3) latency to CR onset, (4) latency to CR peak, (5) latency to UR onset, and (6) latency to first UR peak. For 1, we used all valid trials; for 2–4, we only used the trials in which a CR was present; for 5 and 6, we used US-only trials in the baseline session. Fifth, based on this trial-by-trial analysis, we calculated for each session per mouse the percentage of eyeblink CRs and the mean median, standard deviation, and CV of all other outcome measures. Sixth, statistical effects of session and genotype on all outcome measures were established in R version 1.1.442 using LMMs. All of our outcome measures except CR peak time and CR onset included group, session number (categorical), and their interaction as fixed effects. The random effect included mouse. Data were considered statistically significant if *p* < 0.05.

### Statistics

For plasticity data, the Wilcoxon test (within-group comparisons), and the Mann–Whitney test (between-group comparisons) were used. For rotarod, DigiGait, Sholl analysis, and the electrophysiological characterization shown in Fig 2 (PF strength, PF PPR, cell excitability), repeated-measures ANOVA was used to test differences between genotypes (within-subject factors, respectively: session, limb and speed, radius, stimulus intensity, interpulse interval, current amplitude). For other two-group comparisons (dendritic length, cell density, CF-EPSC), we used an unpaired *t* test. All experiments were done using mutant mice and WT littermate controls in randomized order. In behavioral learning experiments (EBC and VOR gain adaptation), the researchers were blind to mouse genotype. Sample size was determined based on statistical power analysis. Exclusion criteria for cell physiological experiments are stated above. In eye movement recordings, goodness of fits was determined and used for exclusion (R-square value < 0.2), and in case of large differences between double or triple measurements (>30%), lower values were excluded. All values are represented as mean ± SEM, unless stated otherwise.

Data sets are available on open repositories: Dryad (https://doi.org/10.5061/dryad.mh4f7n3) [84] and Neuromorpho.org (RRID: SCR002145) [85].

## Supporting information

S1 Fig Purkinje cell density is normal in SK2-KO mice. Purkinje cell density assessed on sections stained with anti-calbindin antibody (left panel; scale bar: 200 μm) was comparable between SK2-KO mice and WT littermates. Related to Fig 1. KO, knockout; WT, wild type.
(TIF)

S2 Fig Motor coordination and gait are impaired in SK2-KO mice. (A) Walking performance on an accelerating rotating rod (rotarod) was dramatically impaired in SK2-KO: the latency time to fall was virtually null on the first day and improved over time (left panel: latency to fall time averaged per day; right panel: latency to fall time as in the left panel but averaged per session). Control mice include 2 WT and 4 SK2^+/−^ littermates. (B–H) DigiGait analysis of mouse gait on a treadmill at fixed speed was performed at 20 and 25 cm/sec (only 1 out of 11 SK2-KO mice was able to run at 30 cm/s). The bar graphs show a normal stride time (B) and length (D). No alterations were observed in stance width (F). Significant increases were observed in the absolute paw angle (C) and several variability parameters (CV of the stride length [in E], stance width [in G], and the ataxia coefficient [in H]). Overall, these results describe the evident motor impairment that characterizes SK2-KO mice. (I) Cartoon showing sample paw stamps from a control mouse and measured parameters. *p < 0.05, **p < 0.01. Related to Fig 5, S3 Fig, and S1 Table. CV, coefficient of variance; KO, knockout; WT, wild type.
(TIF)

S3 Fig Gait has no sign of tremor or ataxia-like features in L7-SK2 mice. Additional DigiGait results from the experiment reported in Fig 5D and 5E show that differently from SK2-KO mice, L7-SK2 mice had normal paw angle (A), improved stride length (CV) (C), normal stance width (CV) (D), and improved ataxia coefficient (E). Stance width was unaffected by the mutation as in SK2-KO mice (B). *p < 0.05. Related to Fig 5, S2 Fig, and S2 Table. CV, coefficient of variance; KO, knockout.
(TIF)

S1 Table Statistical analysis of DigiGait data of gait in SK2-KO mice. KO, knockout.
(TIF)

S2 Table Statistical analysis of DigiGait data of gait in L7-SK2 mice. (TIF)

S3 Table Statistical analysis of Erasmus Ladder data. (TIF)

S4 Table Compensatory eye movement performance and adaptation analysis. (TIF)

S5 Table Statistical analysis of EBC. EBC, eyeblink conditioning.
(TIF)

## Abbreviations

ACSF: artificial cerebrospinal fluid
AHP: afterhyperpolarization
CF: climbing fiber
CR: conditioned response
CS: conditioned stimulus
CV: coefficient of variance
EBC: eyeblink conditioning
EPSC: excitatory postsynaptic current
EPSP: excitatory postsynaptic potential
FEC: fraction eyelid closure
FLP: flippase
FRT: flippase recognition target
GC: granule cell
ITI: intertrial interval
Kcnn2: potassium calcium-activated channel subfamily N member 2
KO: knockout
LED: light-emitting diode
LMM: linear mixed-effect model
LoxP: locus of X-over of P1
LSD: Least Significant Difference
LTD: long-term synaptic depression
LTP: long-term potentiation
MDMT: magnetic distance measurement technique
MLI: molecular layer interneuron
NVE: NVE Corporation magnetic sensor
OKR: optokinetic response
P: postnatal day
Pcp2: Purkinje cell protein 2
PF: parallel fiber
RT: room temperature
UR: unconditioned response
US: unconditioned stimulus
VOR: vestibulo-ocular reflex
VVOR: visually enhanced VOR
WT: wild type
