# Supplementary figures and images for: SK2 channels in cerebellar Purkinje cells contribute to excitability modulation in motor-learning–specific memory traces
Source: PLoS Biol. 2020 Jan 6;18(1):e3000596. doi: 10.1371/journal.pbio.3000596 (PMC6964916; doi:10.1371/journal.pbio.3000596)

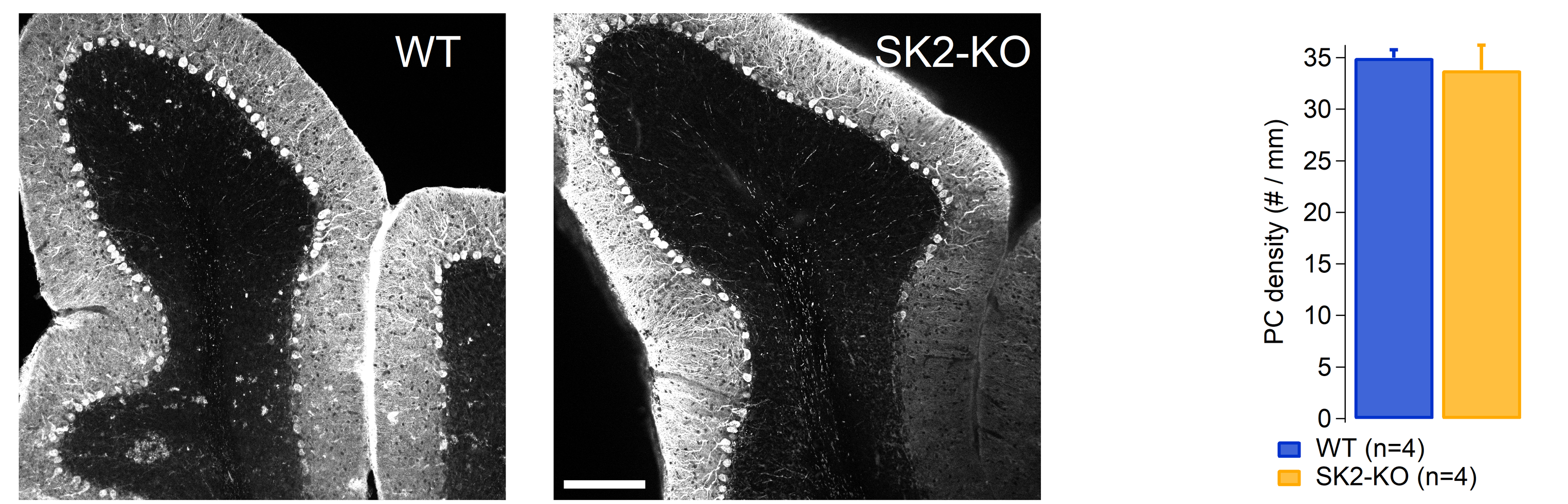

Supplement: S1 Fig — Purkinje cell density assessed on sections stained with anti-calbindin antibody (left panel; scale bar: 200 μm) was comparable between SK2-KO mice and WT littermates. Related to Fig 1. KO, knockout; WT, wild type. (TIF) [file pbio.3000596.s001.tif]

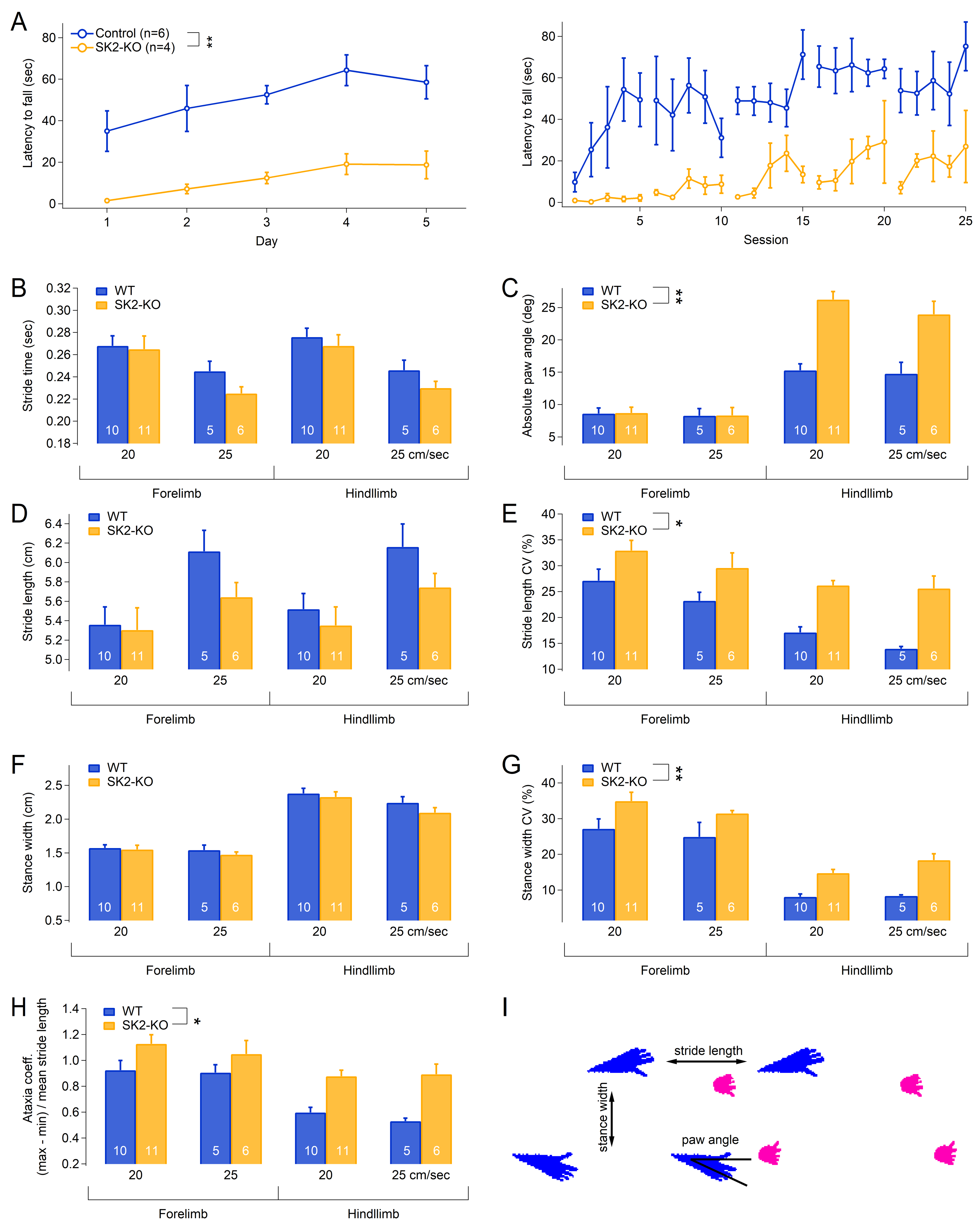

Supplement: S2 Fig — (A) Walking performance on an accelerating rotating rod (rotarod) was dramatically impaired in SK2-KO: the latency time to fall was virtually null on the first day and improved over time (left panel: latency to fall time averaged per day; right panel: latency to fall time as in the left panel but averaged per session). Control mice include 2 WT and 4 SK2+/− littermates. (B–H) DigiGait analysis of mouse gait on a treadmill at fixed speed was performed at 20 and 25 cm/sec (only 1 out of 11 SK2-KO mice was able to run at 30 cm/s). The bar graphs show a normal stride time (B) and length (D). No alterations were observed in stance width (F). Significant increases were observed in the absolute paw angle (C) and several variability parameters (CV of the stride length [in E], stance width [in G], and the ataxia coefficient [in H]). Overall, these results describe the evident motor impairment that characterizes SK2-KO mice. (I) Cartoon showing sample paw stamps from a control mouse and measured parameters. *p < 0.05, **p < 0.01. Related to Fig 5, S3 Fig, and S1 Table. CV, coefficient of variance; KO, knockout; WT, wild type. (TIF) [file pbio.3000596.s002.tif]

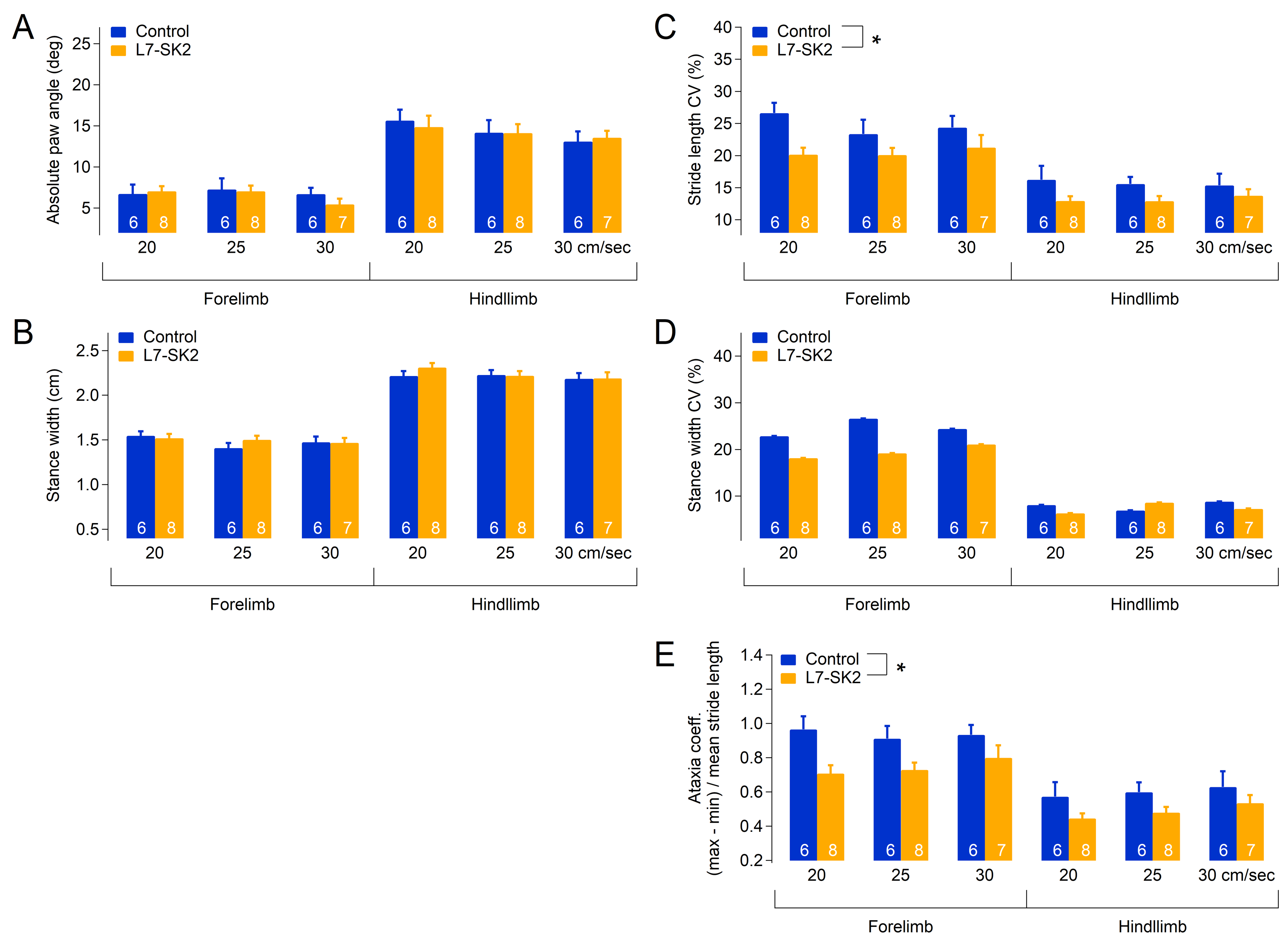

Supplement: S3 Fig — Additional DigiGait results from the experiment reported in Fig 5D and 5E show that differently from SK2-KO mice, L7-SK2 mice had normal paw angle (A), improved stride length (CV) (C), normal stance width (CV) (D), and improved ataxia coefficient (E). Stance width was unaffected by the mutation as in SK2-KO mice (B). *p < 0.05. Related to Fig 5, S2 Fig, and S2 Table. CV, coefficient of variance; KO, knockout. (TIF) [file pbio.3000596.s003.tif]

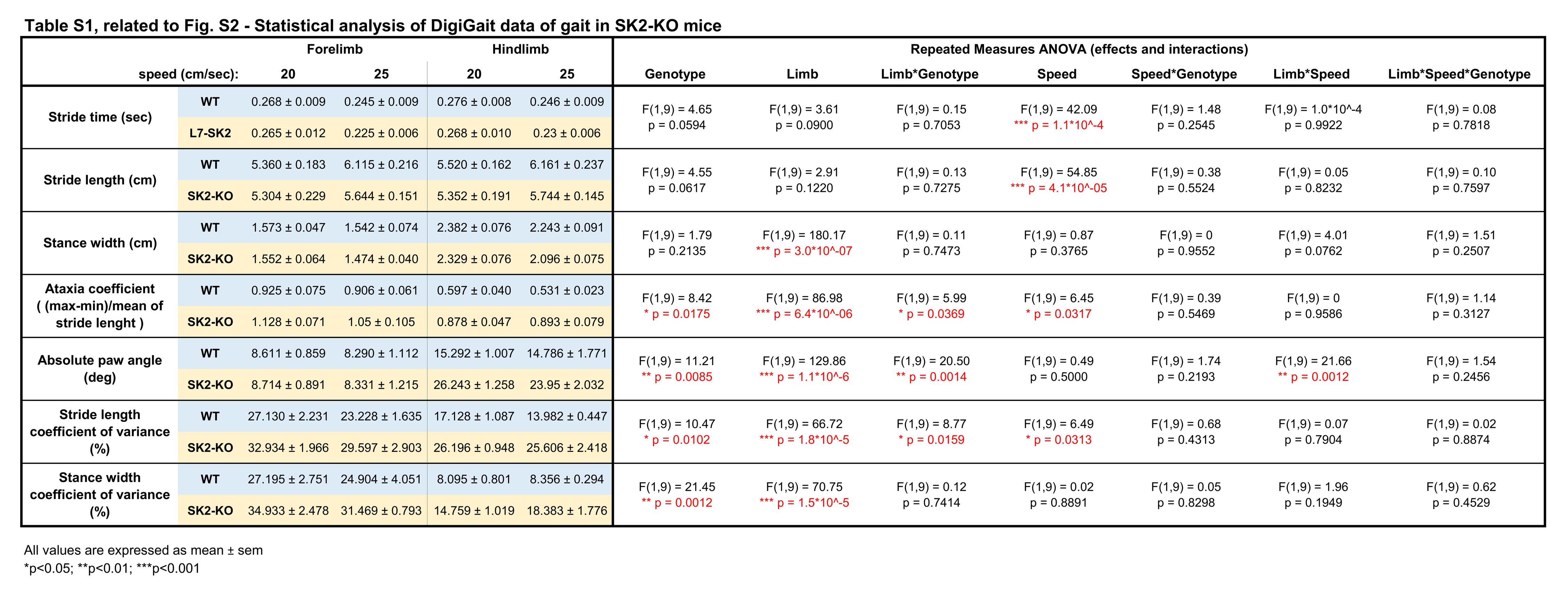

Supplement: S1 Table — KO, knockout. (TIF) [file pbio.3000596.s004.tif]

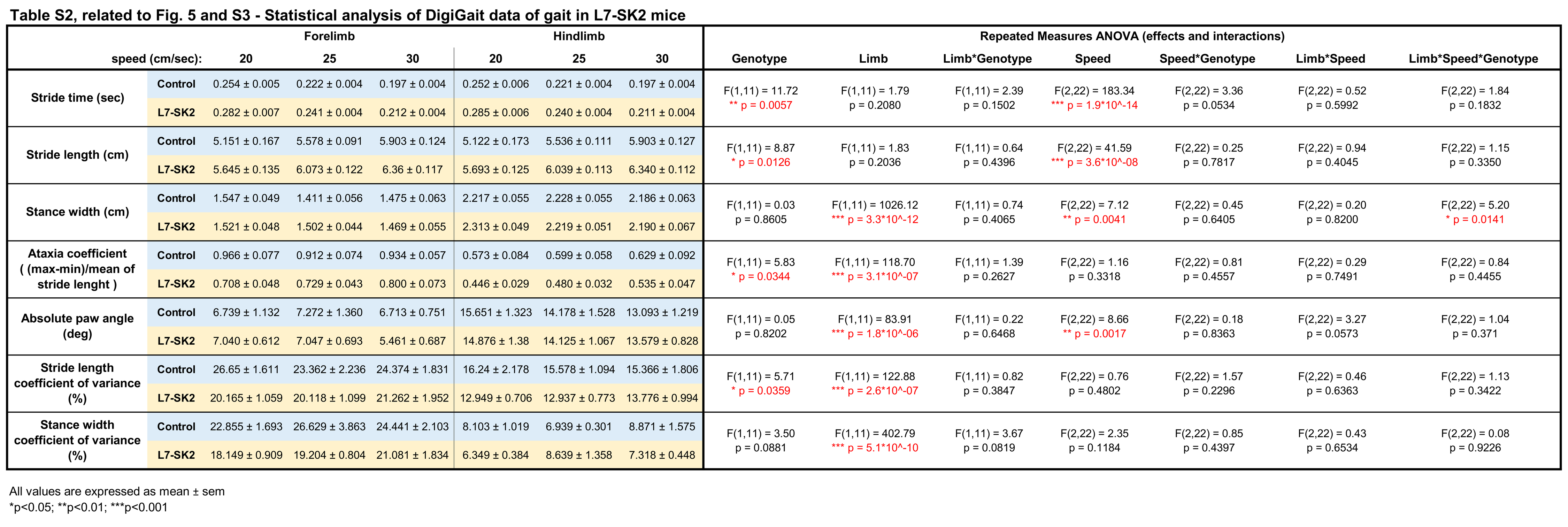

Supplement: S2 Table — (TIF) [file pbio.3000596.s005.tif]

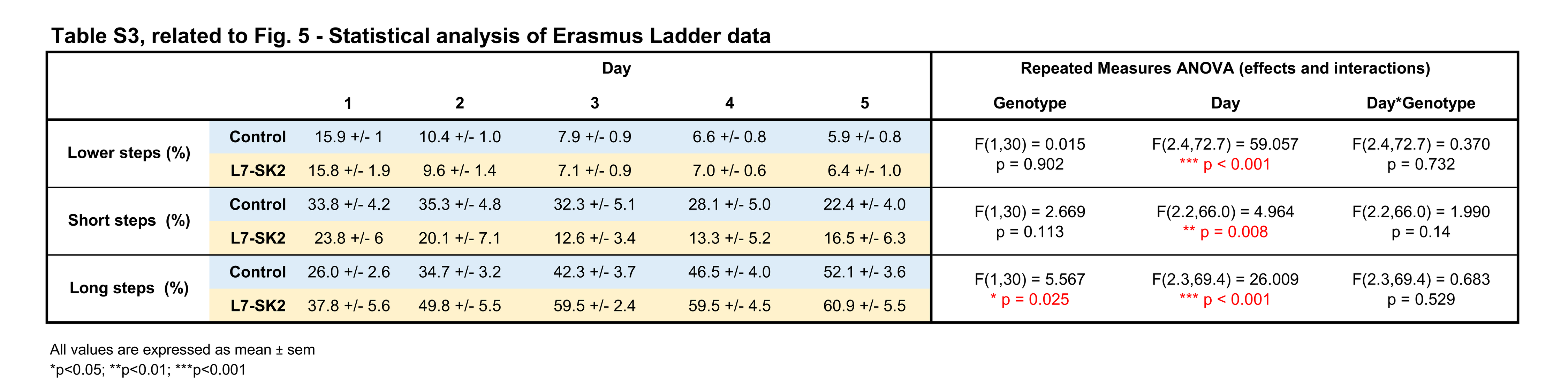

Supplement: S3 Table — (TIF) [file pbio.3000596.s006.tif]

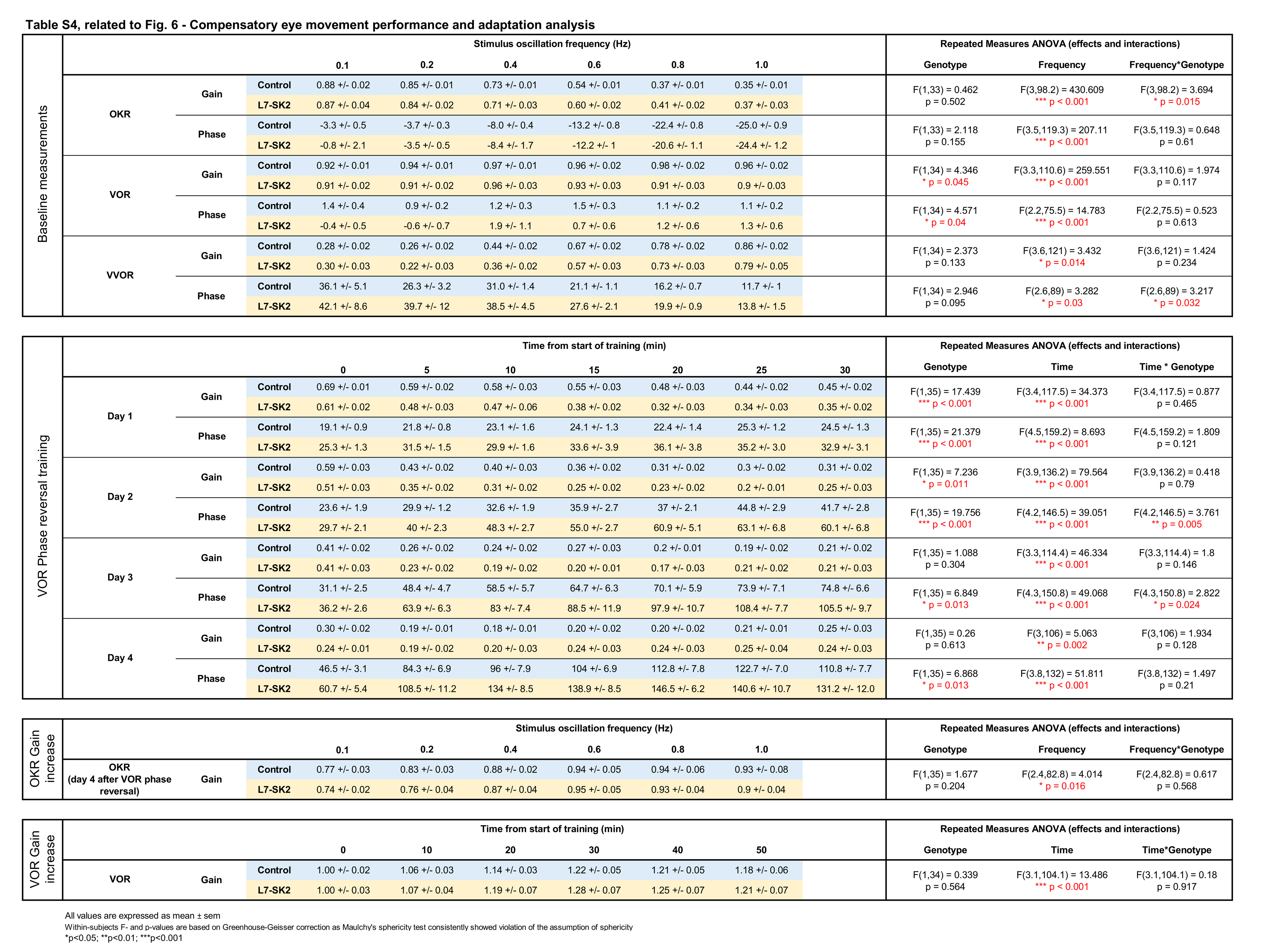

Supplement: S4 Table — (TIF) [file pbio.3000596.s007.tif]

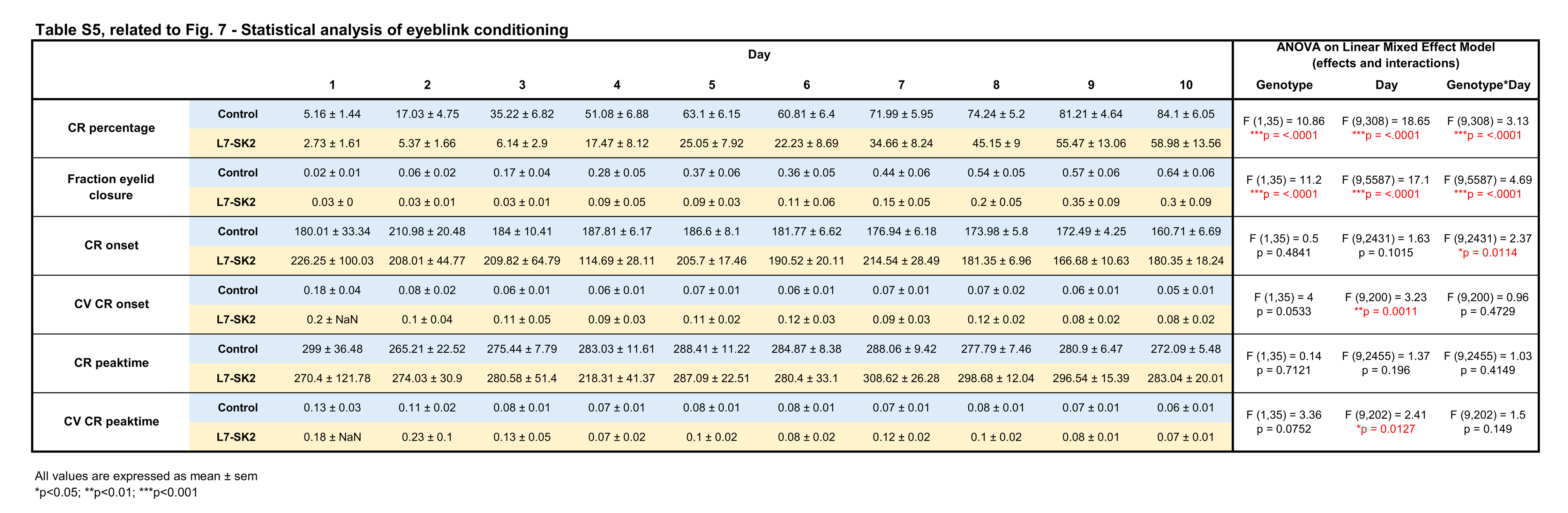

Supplement: S5 Table — EBC, eyeblink conditioning. (TIF) [file pbio.3000596.s008.tif]
